# Genome-wide dissection of globally emergent multi-drug resistant serotype 19A *Streptococcus pneumoniae*

**DOI:** 10.1186/1471-2164-10-642

**Published:** 2009-12-30

**Authors:** Dylan R Pillai, Dea Shahinas, Alla Buzina, Remy A Pollock, Rachel Lau, Krishna Khairnar, Andrew Wong, David J Farrell, Karen Green, Allison McGeer, Donald E Low

**Affiliations:** 1 Department of Laboratory Medicine and Pathobiology, University of Toronto, ON, Canada; 2 Ontario Agency for Health Protection and Promotion, Toronto, ON, Canada; 3 University Health Network/Mount Sinai Hospital, Toronto, ON, Canada

## Abstract

**Background:**

Emergence of multi-drug resistant (MDR) serotype 19A Streptococcus pneumoniae (SPN) is well-documented but causal factors remain unclear. Canadian SPN isolates (1993-2008, n = 11,083) were serotyped and *in vitro *susceptibility tested. A subset of MDR 19A were multi-locus sequence typed (MLST) and representative isolates' whole genomes sequenced.

**Results:**

MDR 19A increased in the post-PCV7 era while 19F, 6B, and 23F concurrently declined. MLST of MDR 19A (*n *= 97) revealed that sequence type (ST) 320 predominated. ST320 was unique amongst MDR 19A in that its minimum inhibitory concentration (MIC) values for penicillin, amoxicillin, ceftriaxone, and erythromycin were higher than for other ST present amongst post-PCV7 MDR 19A. DNA sequencing revealed that alleles at key drug resistance loci *pbp2a*, *pbp2x*, *pbp2b*, *ermB*, *mefA/E*, and *tetM *were conserved between pre-PCV7 ST 320 19F and post-PCV7 ST 320 19A most likely due to a capsule switch recombination event. A genome wide comparison of MDR 19A ST320 with MDR 19F ST320 identified 822 unique SNPs in 19A, 61 of which were present in antimicrobial resistance genes and 100 in virulence factors.

**Conclusions:**

Our results suggest a complex genetic picture where high-level drug resistance, vaccine selection pressure, and SPN mutational events have created a "perfect storm" for the emergence of MDR 19A.

## Background

The introduction of the heptavalent polysaccharide capsule vaccine (PCV7; serotypes 4, 6B, 9V, 14, 18C, 19F, and 23F) in North America as a pediatric universal vaccine program has led to a significant decrease in vaccine serotype invasive pneumococcal disease (IPD) [1] and drug-resistant pneumococci. However, reports of the vaccine's success have been tempered by observed increase in the prevalence of non-vaccine serotypes (NVS) and in particular the multi-drug resistant (MDR) NVS 19A [2-11]. Multi-locus sequence typing (MLST) of these isolates has demonstrated expansion of certain clonal complexes (CC). The genetic determinants that are driving the success of certain CC remain poorly defined. Several plausible and possibly overlapping hypotheses have been suggested. The first suggests that MDR 19A is a result of a genetic recombination event resulting in "capsule switch" [12,13], thereby giving it a fitness advantage over other vaccine serotypes by not being subjected to immune selective pressure. Second, MDR 19A existed before the implementation of PCV7 and has simply replaced vaccine serotypes (VS) targeted by PCV7 [14]. Third, MDR 19A has attained conserved genetic markers which confer resistance to antibiotics commonly used in the treatment of *Streptococcus pneumoniae *(SPN) invasive pneumococcal disease (IPD) [15]. These genetic markers are missing in the drug susceptible SPN 19A isolates, such as those belonging to sequence type 199. Chief amongst these antibiotics conferred resistance to, are β-lactams and macrolides. Resistance to these drugs likely confers a fitness advantage to the organism at the population level where antibiotics are commonly dispensed [15]. Of note, high-level penicillin resistance has been mainly associated with serotypes 6A, 6B, 9V, 14, 19A, 19F, and 23F [1]. Successful expansion of MDR 19A SPN in the post-PCV7 introduction era poses a serious global public health risk. The heptavalent pneumococcal conjugate vaccine (PCV7, Prevnar, Wyeth, Madison, NJ) was approved mid-2001 for use in children in Canada [16]. By early 2006, all provinces and territories in Canada had instituted this vaccine into their routine vaccination programs. We describe here the results of comparative genomics of the emerging multidrug resistant serotype 19A identified from 15 years of SPN serotype and susceptibility testing surveillance in relation to PCV7 introduction in Canada.

## Results

### Serotype surveillance and susceptibility testing of MDR SPN in the Canadian Bacterial Surveillance Network (CBSN) 1993-2008

**Figure 1:**
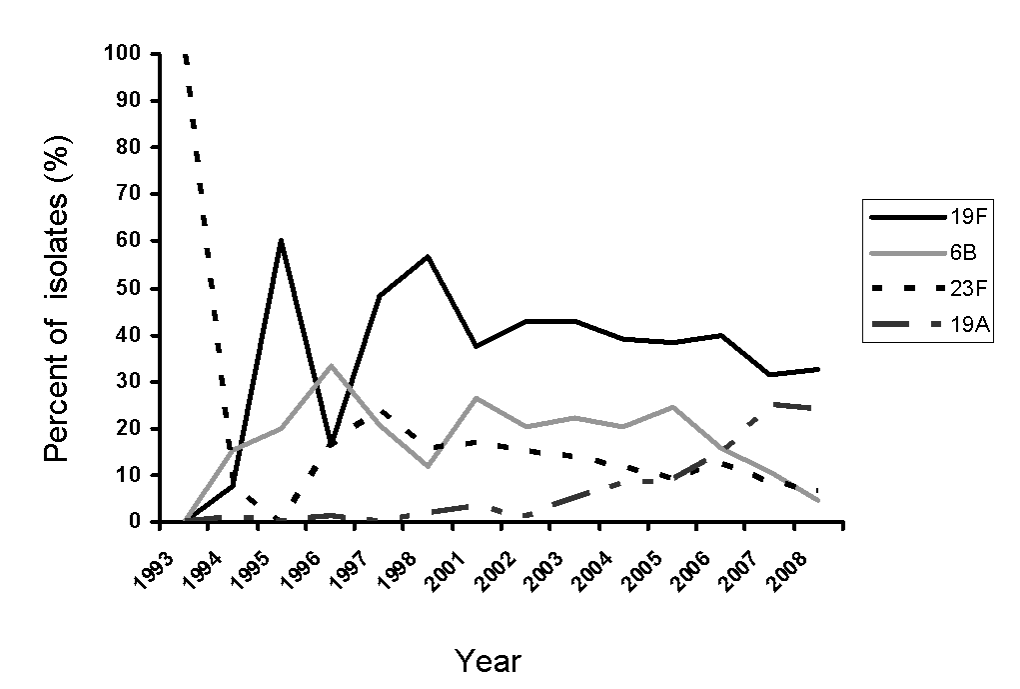
**Serotype trends amongst multi-drug resistant (MDR) strains obtained from the Canadian Bacterial Surveillance Network between 1993 and 2008 (*n *= 11,083)**. MDR 19F (*n *= 477), 23F (*n *= 150), and 6B (*n *= 221) emerged in the pre- PCV 7 introduction era (before 2001) and continued to rise during vaccine introduction (2002-2005), then declined in post-PCV7 introduction era (2006 onwards). MDR 19A (*n *= 97) was present in the pre-PCV7 at very low levels and began to rise soon after PCV7 was introduced country-wide. Data for 1999 and 2000 were not collected. Data are presented as the percent of all MDR isolates collected for the given year.

Serotypes and antibiotic susceptibility testing were determined for *n *= 11,083 isolates over a 15 year period. Serotype surveillance demonstrated a reduction in vaccine serotypes from the era immediately prior to PCV7 introduction in Canada (pre-PCV7, 1993-2001), PCV7 introduction era (2002-2005), and post-PCV7 introduction era (2006-present) (our unpublished data). We focus here on the MDR serotypes. Figure 1 graphically depicts the trends in MDR (defined as non-susceptibility to penicillin plus two other antibiotics) serotypes 6B, 23F, 19F and 19A, the major contributors to MDR SPN in this population as a percent of all MDR isolates collected for that year. While other serotypes contribute to MDR, their numbers were not significantly large in our database.

### Multi-locus sequence typing (MLST) of MDR 19A SPN isolates

**Figure 2:**
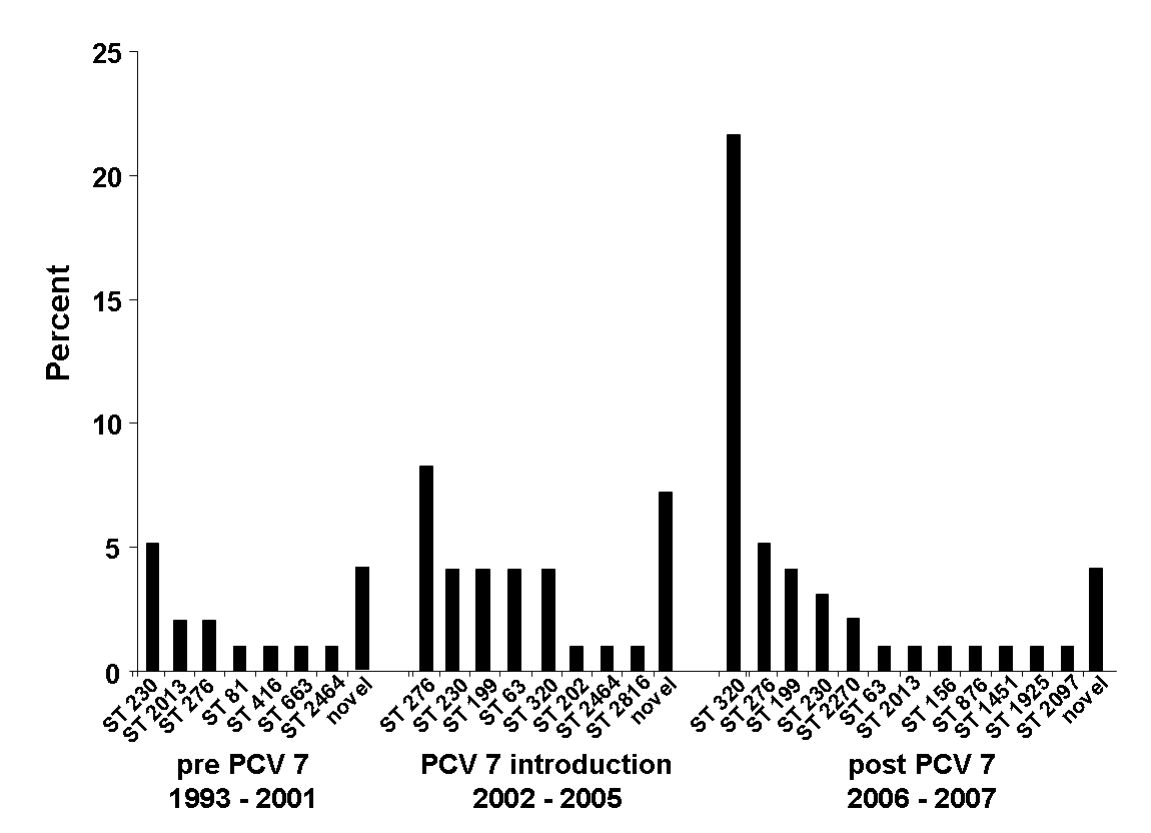
**Multi-locus sequence typing (MLST) of multi-drug resistant (MDR) serotype 19A isolates (n = 97) obtained from the Canadian Bacterial Diseases Surveillance Network during pre-PCV 7 introduction, vaccine introduction, and post-vaccine introduction eras**. Sequence types (ST) are depicted as a percent of all MDR 19 isolates for that period. ST320 has emerged as the singular dominant sequence type amongst MDR 19A isolates in the post-PCV7 era. Novel implies a collection of strains for which no sequence type (ST) was identified within the MLST http://www.mlst.net/ database at this time but submissions have been made and are summarized in Additional file 2. eBURST summary data for MDR 19A, MDR 19F (*n *= 30) and susceptible 19A (*n *= 19) controls are appended in Additional file 1http://www.pillailab.com/suppdata/index.html.

**Figure 3:**
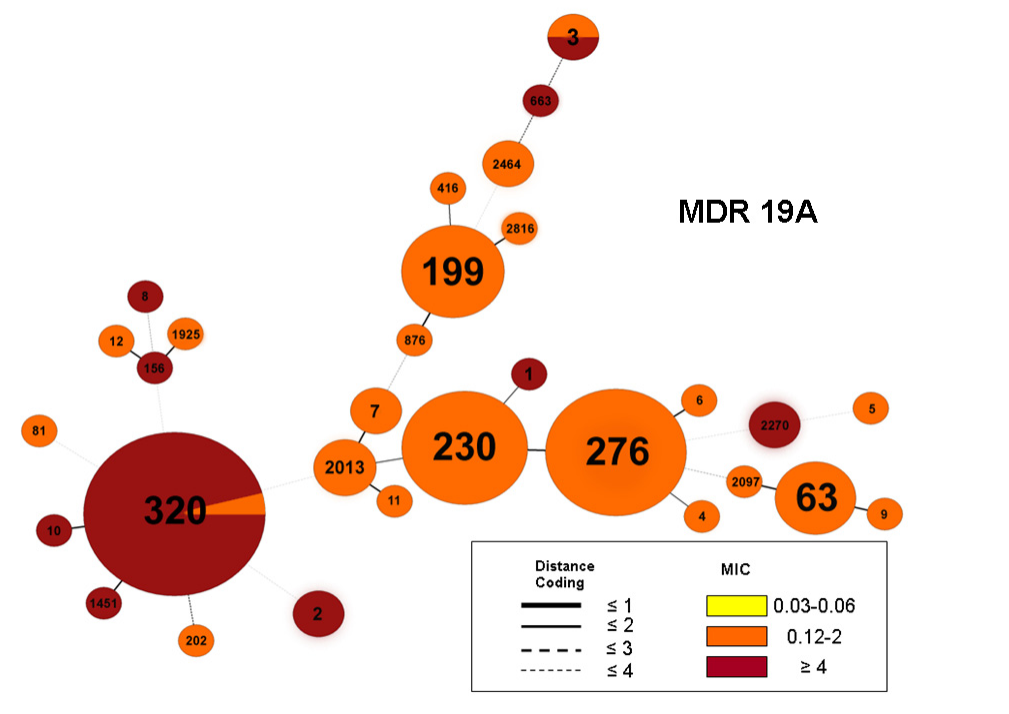
**Minimum spanning tree of multi-drug resistant (A) (MDR) serotype 19A (*n *= 97) using BioNumerics software**. A categorical clustering was performed based on multi-locus sequence type (MLST). Sequence types sharing the maximum number of single-locus variants were connected first. Each circle represents a sequence type (ST) the size of which is proportional to the number of isolates within that particular ST. Colors within circles indicate the minimum inhibitory concentration (MIC) ranges for penicillin. Relationships between the STs are depicted by the lines connecting the STs and the relative lengths of the branches linking them. Distance coding enumerates the number of differences at a given MLST locus. A distance coding of greater than 2 implies a different clonal complex. Angles of the line connections and the overlapping circles have no significance.

**Figure 4:**
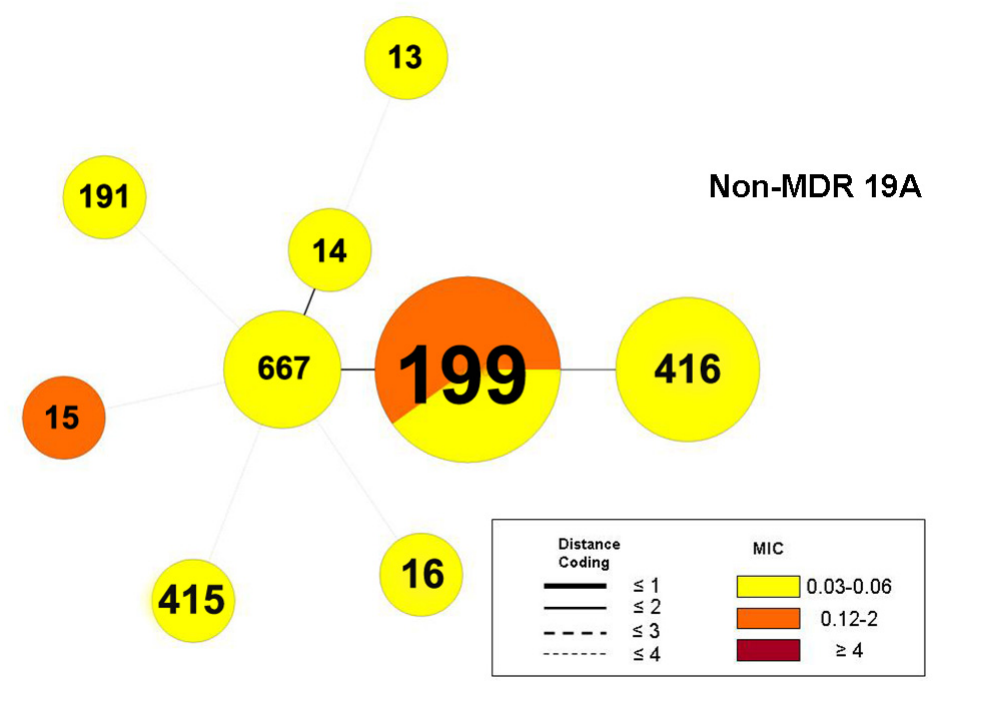
**Minimum spanning tree of multi-drug resistant susceptible 19A control isolates (*n *= 16) using BioNumerics software**. A categorical clustering was performed based on multi-locus sequence type (MLST). Sequence types sharing the maximum number of single-locus variants were connected first. Each circle represents a sequence type (ST) the size of which is proportional to the number of isolates within that particular ST. Colors within circles indicate the minimum inhibitory concentration (MIC) ranges for penicillin. Relationships between the STs are depicted by the lines connecting the STs and the relative lengths of the branches linking them. Distance coding enumerates the number of differences at a given MLST locus. A distance coding of greater than 2 implies a different clonal complex. Angles of the line connections and the overlapping circles have no significance.

**Figure 5:**
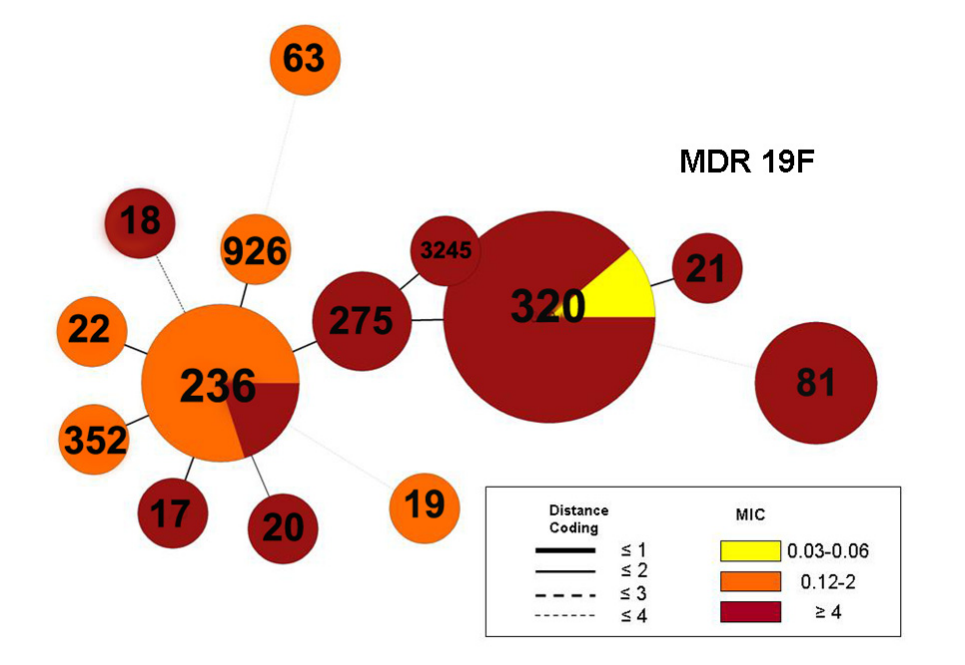
**Minimum spanning tree of multi-drug resistant MDR 19F isolates (*n *= 29) from the pre-PCV7 era using BioNumerics software**. A categorical clustering was performed based on multi-locus sequence type (MLST). Sequence types sharing the maximum number of single-locus variants were connected first. Each circle represents a sequence type (ST) the size of which is proportional to the number of isolates within that particular ST. Colors within circles indicate the minimum inhibitory concentration (MIC) ranges for penicillin. Relationships between the STs are depicted by the lines connecting the STs and the relative lengths of the branches linking them. Distance coding enumerates the number of differences at a given MLST locus. A distance coding of greater than 2 implies a different clonal complex. Angles of the line connections and the overlapping circles have no significance.

Due to the rising absolute number of MDR 19A isolates, MLST was performed to establish the genetic background of these strains. Figure 2 demonstrates that sequence type (ST) 320, part of CC271, accounts for the majority of MDR 19A following the introduction of PCV7 (post-PCV7) in Canada. Prior to PCV7 introduction, ST320 was most significantly associated with serotype 19F in this study (our unpublished data). ST320 is a single-locus variant (different at one gene in the MLST schema comprising seven genes) of Taiwan 19F-14 (ST236) which spread globally in the pre-PCV7 era [17]. Categorical clustering of MDR 19A based on MLST demonstrated that ST320 was associated with high-level penicillin resistance (minimum inhibitory concentration/MIC ≥ 4 μg/mL) (Figure 3). In contrast, non-MDR 19A control isolates taken from CBSN were associated with different STs (Figure 4). Pre-PCV7 MDR 19F high-level penicillin resistance was also strongly associated with ST320 in our Canadian database (Figure 5). Table 1 to 5 summarize actual MIC values for individual MDR 19A isolates by ST. MIC values for penicillin (Table 1), ceftriaxone (Table 2), amoxicillin (Table 3), erythromycin (Table 4), and ciprofloxacin (Table 5) are generally higher for ST320 when compared to other STs amongst MDR 19A. Although not statistically significant, the most notable trends among these antibiotics were penicillin and amoxicillin where ST320 was high-level resistant, while other STs amongst MDR 19A were less resistant or susceptible. Additional file 1http://www.pillailab.com/suppdata/index.html shows eBURST results and summary statistics for all MLST carried out in this study. Novel MLST are detailed in Additional file 2http://www.pillailab.com/suppdata/index.html.

**Table 1:** Penicillin minimum inhibitory concentration (MIC, top row in bold, μg/mL) values for multi-drug resistant (MDR) serotype 19A by sequence type (ST) (*n *= 97).

	S		R					HR	
ST	0.03	0.06	0.12	0.25	0.5	1	2	4	≥ 8
63			4	1					
199	2		11	1					
416	3					1			
230				2	8	2			
276					1	12	2		
**320**							**1**	**22**	**2**
2013				1	2				

S, susceptible; I, intermediate; R, resistant; HR, highly resistant.

**Table 2:** Ceftriaxone minimum inhibitory concentration (MIC, top row in bold, μg/mL) values for multi-drug resistant (MDR) serotype 19A by sequence type (ST) (*n *= 97).

	S					I	R
ST	0.03	0.06	0.12	0.25	0.5	1	≥ 2
63				5			
199	1	4	1	8			
416	3					1	
230			2	9			
276					2	11	2
320					1	1	23
2013				3			

S, susceptible; I, intermediate; R, resistant; HR, highly resistant.

**Table 3:** Amoxicillin minimum inhibitory concentration (MIC, top row in bold, μg/mL) values for multi-drug resistant (MDR) serotype 19A by sequence type (ST) (*n *= 97).

	S								I	R
ST	0.01	0.03	0.06	0.12	0.25	0.5	1	2	4	≥ 8
63			3		2					
199			2	11						
416	1	1	1				1			
230			3	7	1					
230						11	3	1		
**320**								**1**	**1**	**23**
2013				1	1	1				

S, susceptible; I, intermediate; R, resistant; HR, highly resistant.

**Table 4:** Erythromycin minimum inhibitory concentration (MIC, top row in bold, μg/mL) values for multi-drug resistant (MDR) serotype 19A by sequence type (ST) (*n *= 97).

	S				I	R							
ST	0.03	0.06	0.12	0.25	0.5	1	2	4	8	16	32	64	≥ 128
63										1	1	3	
199			5		1			2	4	1	1		
416			4										
230		1	9			1						1	
276											2	12	1
**320**										**1**	**4**	**20**	
2013			3										

S, susceptible; I, intermediate; R, resistant; HR, highly resistant.

**Table 5:** Ciprofloxacin minimum inhibitory concentration (MIC, top row in bold, μg/mL) values for multi-drug resistant (MDR) serotype 19A by sequence type (ST) (*n *= 97).

	S				I	R			
ST	0.12	0.25	0.5	1	2	4	8	16	≥ 32
63			0.5	4			1		
199			4	9	1				
199			2	1	1				
230			1	10					
230				14	1				
**320**			**1**	**23**					**1**
2013			1	2					

S, susceptible; I, intermediate; R, resistant; HR, highly resistant.

### Analysis of antibiotic resistance alleles by DNA sequencing

Mutations associated with elevated minimal inhibitory concentrations (MIC) have been described for key antibiotics commonly used to treat both invasive and non-invasive SPN infection [1,15,18,19]. DNA sequence analysis for key resistance-conferring residues in penicillin binding proteins (*pbp*) *2x*, 1*a*, and *2b *showed conservation between MDR 19A (this study), MDR 19F (this study), and MDR 19A from US and Korea (Table 6). However, residues 339 (F) and 400 (T) of Pbp2x were unique to a representative MDR 19A in the US and have been associated with high-level ceftriaxone resistance not seen in Canadian isolates [10,20]. Susceptible 19A isolates lacked mutations at key residues in *pbp *genes which are associated with β lactam resistance [14]. Similarly, MDR 19A and 19F had complete conservation at residues of *ermB*, *mefA*, *mef E *and *tetM *(all located on the transposon *Tn2010*) associated with resistance to macrolides and tetracyclines [15]. Identical mutations in *Tn2010 *were also observed for representative US and Korea MDR 19A isolates. Genome insertion points of *Tn2010 *were heterogeneous amongst MDR 19A and 19F based on polymerase chain reaction (PCR) using primers derived from whole genome sequencing. Additional file 3http://www.pillailab.com/suppdata/index.html provides the data set for all drug resistance gene sequences and *Tn2010 *insertion site confirmation results by PCR.

**Table 6:** β-lactam resistance genes *pbp1a *(penicillin), *pbp2b *(amoxicillin), and *pbp2x *(ceftriaxone) from reference strain R6 (Genbank AE007317), representative CBSN isolates, US isolates, and South Korean isolates.

	Changes in amino acids of conserved PBP sites									
	pbp 1a			pbp 2x				pbp 2b		
Strain	370-373	428-432	557-559	337-340	394-397	400-401	546-549	385-388	442-445	614-616
19F-14	SAMK		KTG	SAMK	HSSN	MS				
MOH55	SSMK	SRNVT	KTG	SAMK	HSSN	MS	VKSG	SVVK	SSNA	KTGTG
MOH56	SSMK	SRNVT	KTG	SAMK	HSSN	MS	VKSG	SVVK	SSNA	KTGTG
MOH147	STMK	SRNVP	KTG	SAMK	HSSN	MT	LKSG	SVVK	SSNA	KTGTA
MOH83	SSMK	SRNVT	KTG	SAMK	HSSN	MS	VKSG	SVVK	SSNA	KTGTG
MOH62	SSMK	SRNVT	KTG	SAMK	HSSN	MS	VKSG	SVVK	SSNA	KTGTG
Kor914	SSMK	SRNVT	KTG	SAMK	HSSN	MS	VKSG	SVVK	SSNA	KTGTG
Kor21	SSMK	SRNVT	KTG	SAMK	HSSN	MS	VKSG	SVVK	SSNA	KTGTG
Kor39	SSMK	SRNVT	KTG	SAMK	HSSN	MS	VKSG	SVVK	SSNA	KTGTG
PU1175	SSMK	SRNVT	KTG	SAMK	HSSN	MS	VKSG	SVVK	SSNA	KTGTG
PU6055	SSMK	SRNVT	KTG	SAFK	HSSN	TS	VKSG	SVVK	SSNA	KTGTG

Serotype (SERO), sequence type (ST), clonal complex (CC) and MIC values (mg/mL) for penicillin (PEN), ceftriaxone (CTX) and amoxicillin (AMOX) are indicated. Residues associated with resistance to β lactams are in bold letters. Ceftriaxone MIC values for South Korea isolates were unavailable. Of note, Taiwan 19F-14 (ST236) [Genbank E043521/E043522] retains residues similar to 19F and 19A in this study at certain residues of *pbp1a *and *pbp2x*. For the complete data set, including *Tn2010*-related resistance genes, see Additional file 3.

### Whole genome sequence of MDR 19A ST320 from the post-PCV7 introduction era

**Figure 6:**
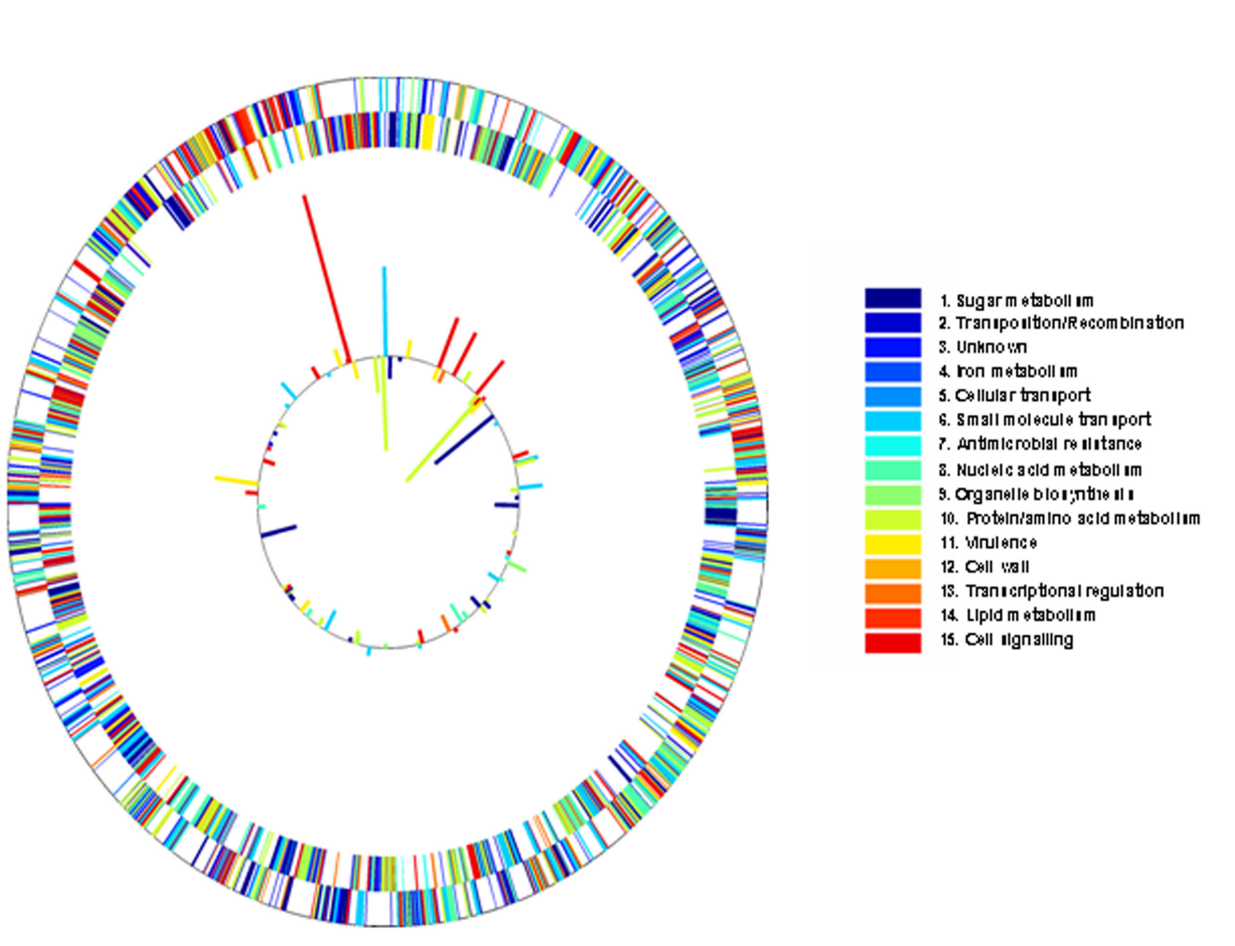
**The whole genome sequence of a representative CBSN isolate of emergent multi-drug resistant serotype 19A ST320 (Genbank Accession GPID ACNU00000000) was compared to a representative isolate of 19F ST320 from the pre-vaccine era (Genbank Accession GPID ACNV00000000)**. The Solexa platform (Illumina Inc, San Diego, CA) was used for sequencing with greater than 100X coverage obtained throughout each genome. Depicted here is the whole genome of a representative MDR 19A ST320 in the post-vaccine era. The locations of proteins encoded on the leading and lagging strands are shown on the outer two rings. Gene ontology categories are color-coded. Of the internal ring, the outermost bars indicate SNPs identified in the leading strand and the innermost bars represent SNP identified in the lagging strand relative to MDR 19F ST320. The length of the bars is propostional to the number of SNPs. For detailed gene identification, location of capsule biosynthetic loci, other key alleles, comparison of 19A and 19F SNPs, as well as a mutation profile compared to reference strain R6 (Genbank AE007317), see Additional file 4http://www.pillailab.com/suppdata/index.html.

A representative isolate of SPN MDR 19A ST320 (Ontario, 2007, sputum isolate from patient with pneumonia) from the period immediately after universal coverage of PCV7 was identified for whole genome sequencing (WGS). Figure 6 summarizes the whole genome and each locus can be navigated in Additional file 4http://www.pillailab.com/suppdata/index.html. MDR 19F ST320 from the pre-PCV7 introduction era (Ontario, 2001, blood isolate from patient with sepsis) was also sequenced using the same method. Sequence comparison of 19F and 19A ST320 genomes, sequenced and identified 0.41% difference in AT content and 0.06% difference in GC content for an overall shared identity of 99.7% between the two genomes. No evidence of large insertions and deletions between the two genomes was identified.

**Figure 7:**
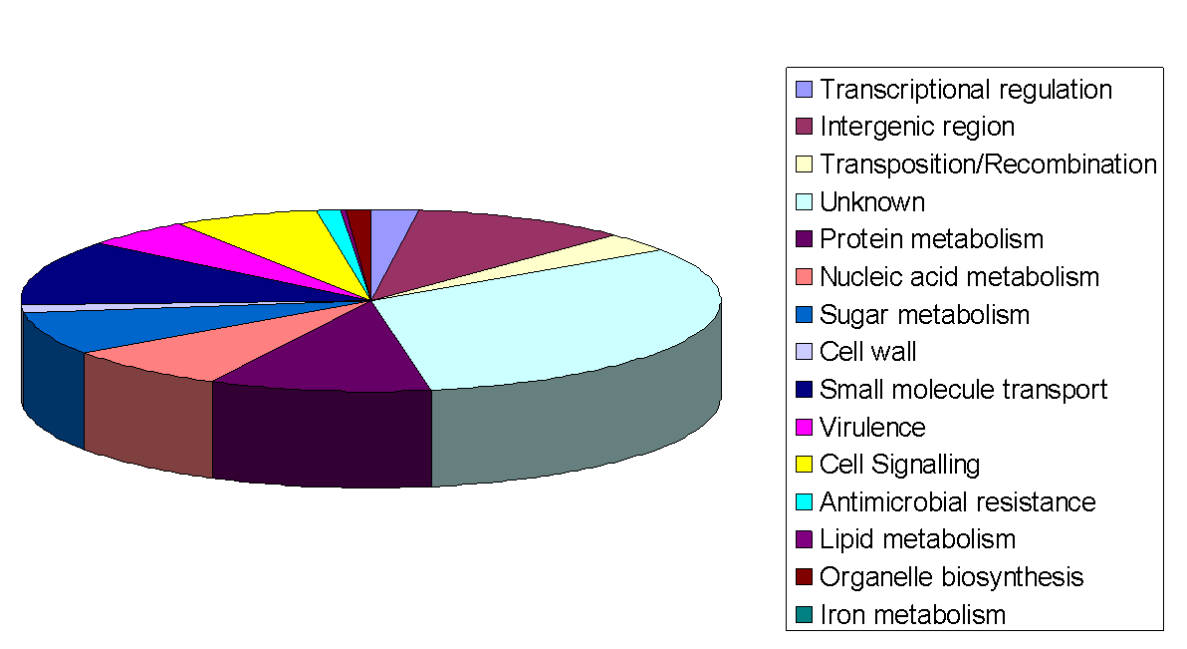
Pie chart percentage breakdown by gene ontology classification (GenoList, Institut Pasteur, Paris) of single nucleotide polymorphisms (SNPs) belonging to MDR 19A ST320 relative to MDR 19F ST320.

Figure 7 provides the breakdown of SNPs unique to MDR 19A ST320. When compared to MDR 19F ST320 and reference strain R6 (Genbank AE007317), 822 unique SNPs were identified in the genome of MDR 19A, 169 of which were non-synonymous [21]. Compared to another serotype 19A WGS in Genbank (Hungary 19A-6, NC_010380, 1989, non-invasive), 9484 SNPs were identified. Of the 822 unique SNPs, 61 SNPs (7.4%) were identified in genes associated with drug resistance and 100 SNPs (12.1%) in virulence factors. A further 14,208 SNPs were shared between MDR 19A and 19F ST320, whereas 1536 SNPs were unique to 19F. Of the 61 unique mutations in antimicrobial resistance genes for MDR 19A, seven were in the *pbp *gene family associated with β lactam resistance, one was in the *crcB *family associated with fluoroquinolone resistance, and 53 were in the folate pathway genes associated with sulfa drug resistance. Unique SNPs in virulence genes included surface protein pspA precursor (*n *= 11 SNPs identified in MDR 19A compared to reference strain R6), determinant for enhanced expression of pheromone (*n *= 4) , hyaluronate lyase precursor (*n *= 4), unsaturated glucuronyl hydrolase (*n *= 2), type 2 capsule locus of SPN (*n *= 39), choline-binding protein F (*n *= 5), choline binding protein A (*n *= 11), histidine kinase (*n *= 3), toxin expression - transcriptional accessory protein (*n *= 4), pneumococcal histidine triad protein D precursor (*n *= 3), immunoglobulin A1 protease (*n *= 11), *N*-acetylneuraminate lyase subunit (*n *= 2), and sialidase B precursor (neuraminidase B) (*n *= 1). Additional file 5http://www.pillailab.com/suppdata/index.html details the gene annotation of unique and shared SNPs (synonymous versus non-synonymous) for MDR 19A and 19F, genome location, and functional category. Summary statistics of the whole genome sequencing can be obtained in Additional files 6 and 7http://www.pillailab.com/suppdata/index.html.

### Capsule locus sequencing analysis

**Figure 8:**
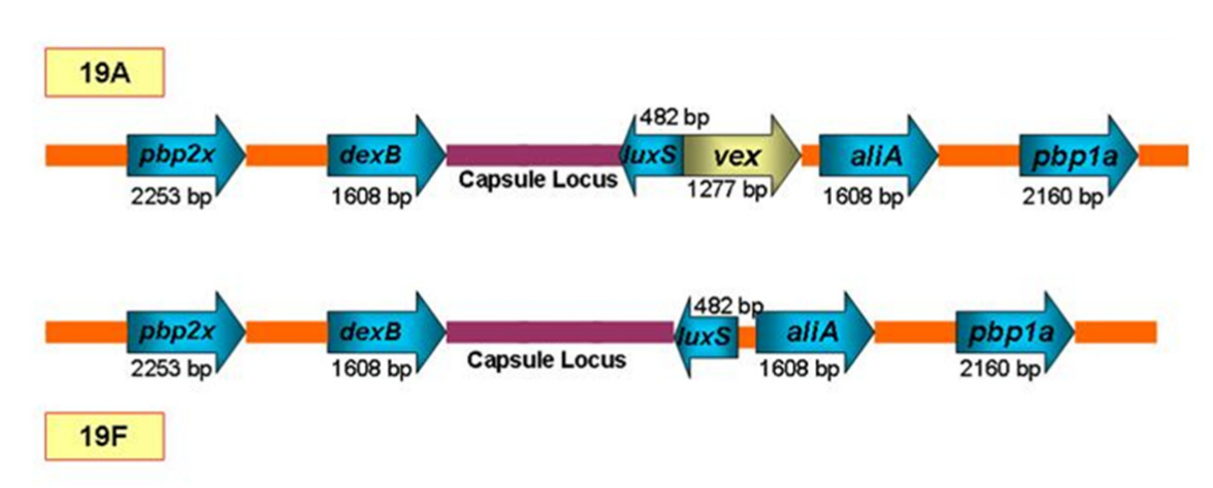
**Alignment of the capsular biosynthetic loci between ST320 MDR19A in the post-PCV7 introduction era and pre-PCV7 MDR19F**. The capsule locus resides between genes *aliA *and *dexB*. The *vex *operon was present directly adjacent to the capsule locus of 19A but not 19F. Of note are the genes *pbp2x *and *pbp1a*, responsible for penicillin resistance, that are present adjacent to the capsule locus. For a full image of the capsule locus and flanking regions please see Additional file 8http://www.pillailab.com/suppdata/index.html.

DNA sequence comparison was carried out for the capsule locus. Figure 8 depicts the arrangement of genes flanking the capsule locus which are similar to previously published data with some notable exceptions [22,23]. For example, A 1277 base pair (bp) region encoding the ABC co-transporter of the *vex *operon was present adjacent to the capsule locus in post-PCV7 MDR 19A ST320 but not in MDR 19F. The *vex *operon in its entirety was located outside of the capsule locus for MDR 19F. The *vex *gene has homology to ABC co-transporters and has been linked to vancomycin tolerance in SPN [24]. Of note, β lactam resistance genes *pbp2x *and *pbp1a *were adjacent to the capsule locus and, as summarized in Table 6, harboured key mutations that confer resistance to β lactam drugs. Alignment of *pbp1a *and *pbp2x *gene sequences from representative ST320 MDR 19A (progeny, *n *= 3), ST320 MDR 19F (putative recipient, *n *= 3), and ST199 19A (putative donor, *n *= 2) demonstrated one recombination point located within *pbp2x *and the other identified by genome walking distal to *pbp1a *(Figure 8 and Additional file 8http://www.pillailab.com/suppdata/index.html). In contrast to *pbp2x *where homologous and heterologous SNPs occur, *pbp1a *showed perfect conservation between recipient and donor strains.

## Discussion

Serotype surveillance of MDR strains in the CBSN database confirmed what other countries have observed - specifically the increased prevalence of MDR 19A, with a concomitant decline in MDR serotypes included in the PCV7 (6B, 23F, and 19F in our study). MLST analysis of MDR 19A was able to identify ST320 as the dominant emerging genotype. This was in contrast to previous studies [10,13] which demonstrated clonal expansion of an existing ST199 MDR 19A after PCV7 introduction but in agreement with other groups [7,8,25]. ST320 appears to have higher than usual resistance to commonly used antibiotics such as penicillin, amoxicillin, and ceftriaxone based on MIC values and is challenging to treat clinically especially in the case of bacterial meningitis [25,5]. This may explain in part ST320 expansion under drug selection pressure. The same genotype and phenotype were present in MDR 19F in the pre-vaccine era - the major contributor to ST320 prior to PCV7 introduction in our study. This was not surprising as 19F ST320 is a single locus variant of Taiwan 19F-14 which spread globally in the pre-PCV7 era [26-28]. It was logical then to test the hypothesis that MDR 19A ST320 emerged from pre-existing MDR 19F ST320. To lend credence to this hypothesis, it was noted early on that PCV7 had differential immunological responses to VS, perhaps allowing certain MDR VS (such as 19F) to survive and co-circulate with non-VS (such as 19A) during PCV7 introduction in Canada [29]. "Capsule switch" has been described previously in SPN and would be the simplest genetic event to account for a 19F to 19A change [12,30].

Brueggemann and colleagues carried out partial DNA sequencing at the capsule locus and demonstrated that recombination points likely lay distal to *pbp1a *and *pbp2x *based on sequence divergence between putative donor, recipient, and progeny strains [12]. This was an important demonstration of capsule switch from vaccine serotype 4 (ST695) to non-vaccine serotype 19A (ST199 and ST695) in the post-PCV7 era [12]. A similar comparison of *pbp1a *and *pbp2x *adjacent to the capsule locus from ST 199 19A (putative donor), ST 320 MDR 19A (putative progeny), and 19F (putative recipient) was undertaken in our study. These data demonstrate definitively that a homologous recombination event has occurred between donor and recipient with breakpoint within *pbp2x *and flanking *pbp1a*, again reinforcing the fact that capsule switch can occur between vaccine and non-vaccine serotypes thereby allowing "vaccine escape". This is a well documented strategy that SPN employs to enhance its fitness [31,32]. Of interest is the presence of the coding sequence of the *vex *operon (containing an efflux pump ABC co-transporter) associated with vancomycin tolerance adjacent to the capsule locus of 19A but located in its entirety outside of this region in 19F. While all strains in this study had an MIC of ≤ 0.5 μg/mL to vancomycin (our unpublished data), it is possible that the dysregulation of *vex*, an ABC-like transporter, by uncoupling it from the rest of the operon, could lead to vancomycin MIC creep. Further evaluation of vancomycin MIC is required to determine the significance of this gene and its location within the capsule amongst MDR strains, especially as vancomycin remains the last line of defense against invasive gram positive infection [5].

In order to dissect the genome further, we undertook WGS comparing a representative isolate of ST320 MDR 19A and 19F. In keeping with the MLST data, near identity (99.7% across the genome) was observed with 14,208 (86%) shared SNPs, when compared to a reference strain R6, between MDR 19A and 19F ST320. Of note, MDR 19A was genetically closer (822 unique SNPs) to MDR 19F in this study than another serotype 19A from Hungary present in GenBank (9484 unique SNPs). These data reinforce that genetic relatedness is better indicated by MLST rather than serotype. Of the 822 unique SNPs identified in MDR 19A, some were in drug resistance markers, virulence factors, cell signaling, and key metabolic genes. Of these, 169 SNPs were non-synonymous. The presence of unique, non-synonymous SNPs inMDR 19A ST320 suggest that unique polymorphisms may also contribute to its success in the post-PCV7 era. The presence of mutations in metabolic genes raises the possibility of increased intrinsic fitness. However, we did not observe significant difference in growth kinetics between various STs associated with MDR 19A in liquid culture experiments (our unpublished data).

South Korea uniquely reported pre-PCV7 MDR 19A ST320. It remains unclear why MDR 19A would emerge without vaccine selection pressure against MDR 19F ST320. Canadian MDR 19A ST320 isolates used in this study were identical at key drug resistance markers to both US and Korean representative isolates suggesting a common source. There was, however, marked heterogeneity of transposon *Tn2010 *insertion sites within a geographic locale suggesting SPN undergoes rapid modification by this mode of genetic change. Furthermore, US isolates had unique mutations which confer high-level resistance to ceftriaxone and have been associated with other ST. Taken together, this suggests that MDR 19A emergence has been caused by distinct genetic events in different geographic locales rather than global spread of a single clone.

## Conclusions

Our data provides evidence that MDR 19A ST320 is genetically derived from MDR 19F ST320 - based on MLST, conservation of SNPs across the genome, key drug resistance markers, and capsule locus structure. However, unique SNPs and heterogeneous transposition events also existed in MDR 19A ST320, suggesting that this strain has adapted and mutated away from a 19F progenitor and is under continuous selection pressure. MLST appears to be limited in explaining the genetic origins of a particular strain as it focuses only on seven housekeeping genes. We confirm that PCV7 vaccine selection pressure, antibiotic selection pressure, and SPN's propensity for genetic change appear to have created a "perfect storm" for MDR 19A emergence. Emergence of genetically heterogeneous MDR 19A appears to be occurring simultaneously in different geographic locales due to similar selection pressures.

Capsule switch events likely occur through genetic transformation in the nasopharynx of children co-infected with different ST of SPN. Of great concern to clinicians is that MDR 19A remains difficult to treat especially in the case of bacterial meningitis where few therapeutic options exist to penetrate the cerebrospinal fluid. If as with methicillin resistance Staphylococcus aureus (MRSA) vancomycin creep occurs (rising MIC values), newer antimicrobial agents will be needed. Furthermore, alternative vaccine strategies that target all serotypes of SPN (protein based vaccines) may be prudent in light of this organism's genetic lability and propensity for vaccine escape. A new 13-valent capsular polysaccharide vaccine (Wyeth, Madison, NJ) is slated for introduction and does include serotype 19A and may forestall its spread. Studies in the developing world are also required to fully understand the extent of the emergence of this strain.

## Methods

### Source of isolates

This work has been approved by the Institutional Review Board of Mt Sinai Hospital, Toronto, Canada. The Canadian Bacterial Surveillance Network is a volunteer group of private and hospital-affiliated laboratories from across Canada which has performed surveillance for antibiotic resistance in Canadian isolates of *S. pneumoniae *since 1988 [33]. Isolates have been provided from a median of 50 laboratories annually which provide service to community and tertiary hospitals, as well as community clinics and doctors' offices. All ten provinces were represented in the sample collection. Laboratories, based on their size and catchment area, were asked to collect either the first 20 or 100 consecutive clinical isolates each year, as well as all sterile site isolates, from 1993 to 2008. In this database, 60% were non-sterile and 40% were sterile. Duplicate isolates from the same patient were excluded.

### Serotype and susceptibility testing

Serotyping was done by the capsular swelling (quellung) test, using Danish antisera (State Serum Institute, Copenhagen, Denmark) [34]. *In vitro *susceptibility testing and interpretation was performed by broth microdilution according to Clinical and Laboratory Standards Institute guidelines [35]. The antimicrobial agents were supplied by their respective manufacturers or were purchased from Sigma (St. Louis, MO.). Multi-drug resistance (MDR) is defined as non-susceptible to penicillin plus any two other classes of antibiotics including macrolides, tetracyclines, fluoroquinolones, or trimethoprim-sulfa.

### Multi-locus sequence typing DNA sequencing, PCR, and whole genome sequencing

MLST was performed according to the standard method described by Spratt and Enright http://www.mlst.net[36]. Briefly, seven housekeeping gene loci were sequenced bidirectionally, uploaded to the MLST website, and analyzed for sequence type and clonal complex associations based on the existing database. DNA sequencing of genes associated with drug resistance (*pbp1a, pbp2b, pbp2x, tetR *and *ermB*) was performed using a standard capillary gene sequencer from Applied Biosystems (Foster City, CA). The Solexa paired-end Sequencing Platform (Illumina, San Diego, CA) was used to generate reads of 50 to 75 bp (with on average greater than 100X coverage for the genome) which were assembled using NextGene (SoftGenetics, State College, PA). [37]. Annotation of the genome was performed using the RAST (Rapid Annotation using Subsystem Technology) server [38]. Additional file 9http://www.pillailab.com/suppdata/index.html summarizes all polymerase chain reaction (PCR) and DNA sequencing primers and associated cycling parameters used in this study to confirm WGS findings at the capsule locus, resistance alleles, and transposon insertion sites.

### Bioinformatic analysis

eBURST analysis of MLST sequence data was performed as described on the MLST website http://www.mlst.net[39]. Genome assembly was performed using NextGene (SoftGenetics, State College, PA) and genome comparison using the Artemis Comparison Tool (Wellcome Trust Sanger Institute, Cambridge, UK). Gene ontology classification was achieved using GenoList (Institut Pasteur, Paris). Gene ontology categories were identified using AmiGO [21].

### Data Submission and Analysis

This Whole Genome Shotgun project has been deposited at DDBJ/EMBL/GenBank under the accession ACNU00000000 (*Streptococcus pneumoniae *str. Canada MDR_19A identified in Ontario, Canada in 2007) and ACNV00000000 (*Streptococcus pneumoniae *str. Canada MDR_19F identified in Ontario, Canada in 2001). This paper describes the first versions of the whole genome for Canada MDR_19A (ACNU10000000) and MDR_19F (ACNV10000000). Detailed statistics about the genome sequences and the assembly, coverage and distribution reports as well as a summary of the SNP profiles is provided in Additional files 6 and 7. Novel MLST allele submission numbers are summarized in Additional file 2.

## Authors' contributions

DRP and DS designed the study, analyzed the data, and wrote the manuscript. AB and RAP carried out data analysis and technical work. KK, AW, DJF, AM, KG, and DEL contributed to data analysis and manuscript review. DEL, AM, and KG, were responsible for providing the isolates, data anaylsis and manuscript review. All authors agree with the current version of the manuscript.

## Supplementary Material

Additional file 1 **The complete MLST data set, summary statistics and eBURST figures**. http://www.mlst.net. eBURST figures can be obtained by clicking on the hyperlinks. Figures are presented for MDR 19A, control 19A and 19F isolates referred to in the text, as well as isolates based on era (pre-PCV7, PCV7, and post-PCV7). All supplementary data can also be accessed at the following website http://www.pillailab.com/suppdata/index.html Click here for file

Additional file 2 New MLST sequence type and submission numbers identified in this study. Click here for file

Additional file 3 **Complete data set for β lactam (*pbp*), erythromycin (*erm*), and tetracycline (*tet*) resistance gene mutation alleles**. Sequence analysis of Tn2010 insertion sites was based on primers designed from the WGS. A (+) indicated amplification and (-) no amplification by PCR for each isolate tested using primers derived from the WGS. These data demonstrated heterogeneity amongst MDR ST320 19A and MDR 19F isolates in the pre-PCV7 era with some common insertion points between serotypes. Tn2010 constituent genes *ermB*, *mefA*, *tetM*, and *mega *were highly conserved amongst post-PCV7 MDR 19A and pre-PCV7 MDR 19F. Click here for file

Additional file 4 **The annotated whole genome sequence data set for representative isolates of MDR 19A ST320**. The first tab shows the full gene listing. To zoom in on a portion of the genome, click on the circular genome at the location of interest. The lower panel of the first tab depicts the entire capsule locus and flanking genes. The second tab gives the full mutation profile. The third tab gives the functional categories present in the genome. Click here for file

Additional file 5 **Mutation report of all single nucleotide polymorphisms, genomic location, and their gene ontology classification**. Specific data for MDR 19F (tab 1), 19A (tab 2), and both (tab 3), relative to reference strain R6 (Genbank AE007317) are shown. Unique SNPs for 19F (tab 4) and 19A (tab 5) and subsequent analysis (tab 6) are alsolisted. Click here for file

Additional file 6 Whole genome statistics, SNP summary, distribution and coverage report of the genomes for MDR 19A Click here for file

Additional file 7 Whole genome statistics, SNP summary, distribution and coverage report of the genomes for MDR 19A Click here for file

Additional file 8 **Sequence alignment of *pbp1a *and *pbp2x *for putative donor strain ST199 19A, putative recipient strain ST320 MDR 19F, and progeny strain ST320 MDR 19A**. Yellow highlights sequence conservation between two of the three strain types. An arrow indicates the recombination point for *pbp2x *where the conservation switches between strain types suggesting a recombination point. For *pbp1a *no recombination point is identified within the gene suggesting that the point lies outside the gene. Heterologous and homologous single nucleotide polymorphisms (SNPs) are also present within *pbp2x *but not *pbp1a *(marked by arrows). A figure summarizing the genetic recombination event between donor, recipient and progeny strains is shown. Click here for file

Additional file 9 Complete set of primers and cycling conditions used for polymerase chain reaction (PCR) or DNA sequencing in this study. Click here for file
