# Supplementary material for: Genome-wide dissection of globally emergent multi-drug resistant serotype 19A Streptococcus pneumoniae
Source: BMC Genomics. 2009 Dec 30;10:642. doi: 10.1186/1471-2164-10-642 (PMC2807444; doi:10.1186/1471-2164-10-642)

***Streptococcus pneumoniae* MDR 19F Genome Statistics** Summary

| **Feature** | **Description** |
| --- | --- |
| Sequencing | Paired reads – read length: 36 |
| Reference genome | R6 |
| Assembly | Alignment, *De Novo* (De Bruijn) |
| Coverage | *Please, see diagrams below* |
| Short reads not assembled into contigs (<100bp) | 475 |
| Annotation of the unassembled short reads | Batch BLAST and tblastx. (If classified as Intergenic, genomic match was found, but no ORF match found). Most belong to transposases, intergenic regions or unknown function. |
| Number of Contigs (>100bp) | 277 |
| Number of bases | 1986627 |
| GC Content | 39.7 |
| Minimum contig length | 372bp |
| Median contig length | 4718bp |
| Mean contig length | 7172bp |
| Maximum contig length | 41516bp |
| Annotation of the Contigs | RAST (Rapid Annotations using Subsystems Technology; Aziz et al, BMC Genomics 2008) |
| Number of Single Nucleotide Polymorphisms relative to R6 | 15744 (relative to the genome of *Streptococcus pneumoniae* R6) |
| Number of Single Nucleotide Polymorphisms in the 19F genome (not found in MDR 19A) | 1536 |
| Number of Non-Synonymous Single Nucleotide Polymorphisms | 168 |

**Distribution Report**


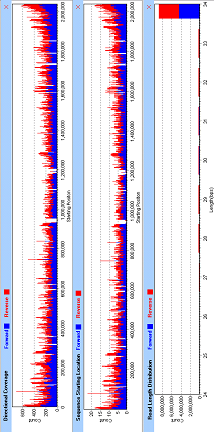


**Coverage Report**


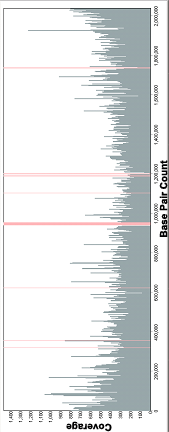

Supplement: Additional file 7 — Whole genome statistics, SNP summary, distribution and coverage report of the genomes for MDR 19A [file 1471-2164-10-642-S7.DOC]
