# Supplementary material for: Genome-wide dissection of globally emergent multi-drug resistant serotype 19A Streptococcus pneumoniae
Source: BMC Genomics. 2009 Dec 30;10:642. doi: 10.1186/1471-2164-10-642 (PMC2807444; doi:10.1186/1471-2164-10-642)
Supplement: Additional file 8 — Sequence alignment of pbp1a and pbp2x for putative donor strain ST199 19A, putative recipient strain ST320 MDR 19F, and progeny strain ST320 MDR 19A. Yellow highlights sequence conservation between two of the three strain types. An arrow indicates the recombination point for pbp2x where the conservation switches between strain types suggesting a recombination point. For pbp1a no recombination point is identified within the gene suggesting that the point lies outside the gene. Heterologous and homologous single nucleotide polymorphisms (SNPs) are also present within pbp2x but not pbp1a (marked by arrows). A figure summarizing the genetic recombination event between donor, recipient and progeny strains is shown. [file 1471-2164-10-642-S8.DOC]

MUSCLE (3.7) multiple sequence alignment


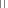


**D:19F**


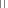

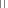

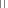

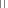


**R:19A ST199**


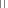


**P:19A ST320**

***pbp2x***

***dexB***

***AliA***

***pbp1a***

**n=3**

**n=3**

**n=3**

**Capsular Locus**

Heterologous SNP to donor and recipient

Homologous SNP to either donor or recipient

Recombination point where homologous SNPs switch between recipient and donor

19A ST199 (1) *pbp2x* TAGTCTCCTAAAGTTAATGTAATTTTTTTAATGTCCTTGATAGCTGTATTAGCACGAACATCTTGCTTCTGCACAGTAGTGCCCGAACCTTCAAATTCAAGTTCTATATTGAGCCACTTAGCAAAGGTCTCAGCAGTCGCCTTTGTCCAACCATACATGTCTGGAACTTCCTCTGCTTT

19A ST199 (2) *pbp2x* TAGTCTCCTAAAGTTAATGTAATTTTTTTAATGTCCTTGATAGCTGTATTAGCACGAACATCTTGCTTCTGCACAGTAGTGCCCGAACCTTCAAATTCAAGTTCTATATTGAGCCACTTAGCAAAGGTCTCAGCAGTCGCCTTTGTCCAACCATACATGTCTGGAACTTCCTCTGCTTT

19F ST320 (1) *pbp2x* TAGTCTCCTAAAGTTAATTTAATTTTTTTAATGTTTTTGATAGCTGTATTAGTCCGAACATCTTGCTTCTGAACAACGGAACCTGAACCTTCAAATTCCAGTTCAATATCCAACCATTTAGCAAAGGTCTCGGCAGTCTCTTTTTTCCAGCCATACATGTCTGGAATTTCTTCTACCTT

19F ST320 (2) *pbp2x* TAGTCTCCTAAAGTTAATTTAATTTTTTTAATGTTTTTGATAGCTGTATTAGTCCGAACATCTTGCTTCTGAACAACGGAACCTGAACCTTCAAATTCCAGTTCAATATCCAACCATTTAGCAAAGGTCTCGGCAGTCTCTTTTTTCCAGCCATACATGTCTGGAATTTCTTCTACCTT

19F ST320 (3) *pbp2x* TAGTCTCCTAAAGTTAATTTAATTTTTTTAATGTTTTTGATAGCTGTATTAGTCCGAACATCTTGCTTCTGAACAACGGAACCTGAACCTTCAAATTCCAGTTCAATATCCAACCATTTAGCAAAGGTCTCGGCAGTCTCTTTTTTCCAGCCATACATGTCTGGAATTTCTTCTACCTT

19A ST320 (1) *pbp2x* TAGTCTCCTAAAGTTAATGTAATTTTTTTAATGTCCTTGATAGCTGTGTTAGCACGAACATCTTGCTTCTGCACAGTAGAACCTGAACCTTCAAATACAAGTTCTATATTTAACCACTTAGAAAAAGCTTCTGCGGTTGCTTTTGTCCAACCATACATATCTGGAATTTCTTCTACCTT

19A ST320 (2) *pbp2x* TAGTCTCCTAAAGTTAATGTAATTTTTTTAATGTCCTTGATAGCTGTGTTAGCACGAACATCTTGCTTCTGCACAGTAGAACCTGAACCTTCAAATACAAGTTCTATATTTAACCACTTAGAAAAAGCTTCTGCGGTTGCTTTTGTCCAACCATACATATCTGGAATTTCTTCTACCTT

19A ST320 (3) *pbp2x* TAGTCTCCTAAAGTTAATGTAATTTTTTTAATGTCCTTGATAGCTGTGTTAGCACGAACATCTTGCTTCTGCACAGTAGAACCTGAACCTTCAAATACAAGTTCTATATTTAACCACTTAGAAAAAGCTTCTGCGGTTGCTTTTGTCCAACCATACATATCTGGAATTTCTTCTACCTT

****************** *************** *********** **** ***************** *** * ** ************ * ***** **** * *** **** *** * ** ** ** * *** **** ******** ******* *** *** * **

19A ST199 (1) *pbp2x* ATCAGATAAGATCAGGACTTGCTGATTCGGGGCAAGATTCTTCCCTTCTTCAGCAGAACTGTTTTTAATCTTCGTTCCTGTTCCCACAACAATAGGTTGTACAAGATTGCGACGCAATTCTTCTGCTAAATCACCAGGTGAAATATCCTTGACACTAGGCATAGGATAAG--GACTTTGT

19A ST199 (2) *pbp2x* ATCAGATAAGATCAGGACTTGCTGATTCGGGGCAAGATTCTTCCCTTCTTCAGCAGAACTGTTTTTAATCTTCGTTCCTGTTCCCACAACAATAGGTTGTACAAGATTGCGACGCAATTCTTCTGCTAAATCACCAGGTGAAATATCCTTGACACTAGGCATAGGATAAG--GACTTTGT

19F ST320 (1) *pbp2x* ATCCGATAAAAGGAGAACTTGTTGGTTTGGTGCAAGATTGGTCCCTTCTTCTACAGAAGTCTCTTTAATCTTTGTTCCAGTACCTACAACGATTGGTTGCACAATATTTCGGCGTAAGGCTTCCGCCAACTCACCAGGTGAAATATCCTTGATGCTAGGCATTGCATAAGAAGATTCTGT

19F ST320 (2) *pbp2x* ATCCGATAAAAGGAGAACTTGTTGGTTTGGTGCAAGATTGGTCCCTTCTTCTACAGAAGTCTCTTTAATCTTTGTTCCAGTACCTACAACGATTGGTTGCACAATATTTCGGCGTAAGGCTTCCGCCAACTCACCAGGTGAAATATCCTTGATGCTAGGCATTGCATAAGAAGATTCTGT

19F ST320 (3) *pbp2x* ATCCGATAAAAGGAGAACTTGTTGGTTTGGTGCAAGATTGGTCCCTTCTTCTACAGAAGTCTCTTTAATCTTTGTTCCAGTACCTACAACGATTGGTTGCACAATATTTCGGCGTAAGGCTTCCGCCAACTCACCAGGTGAAATATCCTTGATGCTAGGCATTGCATAAGAAGATTCTGT

19A ST320 (1) *pbp2x* ATCCGATAAAAGGAGAACTTGTTGGTTTGGTGCAAGATTTTTTCCTTCTTCTACAGAAGTCTCTTTAATCTTTGTTCCAGTACCAACAACGATTGGTTGCACAATATTTCGACGTAAGGCTTCCGCCAAATCACCAGGTGAAATATCCTTGATGCTAGGCATTGCATACGAAGATTCTGT

19A ST320 (2) *pbp2x* ATCCGATAAAAGGAGAACTTGTTGGTTTGGTGCAAGATTTTTTCCTTCTTCTACAGAAGTCTCTTTAATCTTTGTTCCAGTACCAACAACGATTGGTTGCACAATATTTCGACGTAAGGCTTCCGCCAAATCACCAGGTGAAATATCCTTGATGCTAGGCATTGCATACGAAGATTCTGT

19A ST320 (3) *pbp2x* ATCCGATAAAAGGAGAACTTGTTGGTTTGGTGCAAGATTTTTTCCTTCTTCTACAGAAGTCTCTTTAATCTTTGTTCCAGTACCAACAACGATTGGTTGCACAATATTTCGACGTAAGGCTTCCGCCAAATCACCAGGTGAAATATCCTTGATGCTAGGCATTGCATACGAAGATTCTGT

*** ***** * ** ***** ** ** ** ******** * ******** ***** * * ********* ***** ** ** ***** ** ***** **** *** ** ** ** **** ** ** ********************** ******** * *** * ** * ***

19A ST199 (1) *pbp2x* TGACTTACTTGCTCCAAAGATTTAGCTGTTGTTTGAAGATTGAGAGAGTCTTTCATAGCTGAAGCCCGCTCCAAGATAGGATTGGCAAATTCTCCCAACTGAATACCTGAATAATGTTCAGGTTGTTGGACCGTCACATACAAGATAAAATCAGGATTTTCAGCCGGACTCATCGATACA

19A ST199 (2) *pbp2x* TGACTTACTTGCTCCAAAGATTTAGCTGTTGTTTGAAGATTGAGAGAGTCTTTCATAGCTGAAGCCCGCTCCAAGATAGGATTGGCAAATTCTCCCAACTGAATACCTGAATAATGTTCAGGTTGTTGGACCGTCACATACAAGATAAAATCAGGATTTTCAGCCGGACTCATCGATACA

19F ST320 (1) *pbp2x* CG--TAACTTTATCTAAATTTTTGGCTGGAGATTGAAGATTGAGAGATTCTTTCATAGCTGAAGCCCGCTCCAAGATTGGGGTGGCAAATTCTCCCAACTGGATACCTGAATAATGCTCAGGCTGTTGAACCGTTACATACAAGATAAAATCAGGATTTTCAGCAGGATTCATAGTCACA

19F ST320 (2) *pbp2x* CG--TAACTTTATCTAAATTTTTGGCTGGAGATTGAAGATTGAGAGATTCTTTCATAGCTGAAGCCCGCTCCAAGATTGGGGTGGCAAATTCTCCCAACTGGATACCTGAATAATGCTCAGGCTGTTGAACCGTTACATACAAGATAAAATCAGGATTTTCAGCAGGATTCATAGTCACA

19F ST320 (3) *pbp2x* CG--TAACTTTATCTAAATTTTTGGCTGGAGATTGAAGATTGAGAGATTCTTTCATAGCTGAAGCCCGCTCCAAGATTGGGGTGGCAAATTCTCCCAACTGGATACCTGAATAATGCTCAGGCTGTTGAACCGTTACATACAAGATAAAATCAGGATTTTCAGCAGGATTCATAGTCACA

19A ST320 (1) *pbp2x* CG--TCACTTGATCTAAATTCTTAGCTGGAGATTGAAGATTGAGAGATTCTTTCATAGCTGAAGCCCGCTCCAAGATTGGGGTGGCAAATTCTCCCAACTGGATACCTGAATAATGCTCAGGCTGTTGAACCGTTACATACAAGATAAAATCAGGATTTTCAGCAGGATTCATAGTCACA

19A ST320 (2) *pbp2x* CG--TCACTTGATCTAAATTCTTAGCTGGAGATTGAAGATTGAGAGATTCTTTCATAGCTGAAGCCCGCTCCAAGATTGGGGTGGCAAATTCTCCCAACTGGATACCTGAATAATGCTCAGGCTGTTGAACCGTTACATACAAGATAAAATCAGGATTTTCAGCAGGATTCATAGTCACA

19A ST320 (3) *pbp2x* CG--TCACTTGATCTAAATTCTTAGCTGGAGATTGAAGATTGAGAGATTCTTTCATAGCTGAAGCCCGCTCCAAGATTGGGGTGGCAAATTCTCCCAACTGGATACCTGAATAATGCTCAGGCTGTTGAACCGTTACATACAAGATAAAATCAGGATTTTCAGCAGGATTCATAGTCACA

* * **** ** *** ** **** * *************** ***************************** ** ******************* ************** ***** ***** ***** ***************************** *** **** * ***

19A ST199 (1) *pbp2x* GCCGAAAAAATATAGTTAGTTAACCCGACTAGATAACCACCATTTTTCTCGTCAGCAATCTGAGCCGTACCAGACTTGAGGGCTACATTTTGCCCAGGAACAGTTACAGTTGGCTTGCCTGTGCTGTGGTTATACATGGTTCCATAAACCGGATCCGTCCCTACCAAAACCATGTTAGTC

19A ST199 (2) *pbp2x* GCCGAAAAAATATAGTTAGTTAACCCGACTAGATAACCACCATTTTTCTCGTCAGCAATCTGAGCCGTACCAGACTTGAGGGCTACATTTTGCCCAGGAACAGTTACAGTTGGCTTGCCTGTGCTGTGGTTATACATGGTTCCATAAACCGGATCCGTCCCTACCAAAACCATGTTAGTC

19F ST320 (1) *pbp2x* GCTGAGAAAATATAATTGGTAGAACCAACCAAGTATCCTCCATTTTTCTCATCAGCGATTTGAGCCGTACCGGATTTAACTGCTACATTTTGTCCAGGAACTGTTATAATTGGCTTTCCTGTGTAGTGATTATACATAGTTCCATATAGAGGGTCCGTCCCAACTAATATCATGTTAGTA

19F ST320 (2) *pbp2x* GCTGAGAAAATATAATTGGTAGAACCAACCAAGTATCCTCCATTTTTCTCATCAGCGATTTGAGCCGTACCGGATTTAACTGCTACATTTTGTCCAGGAACTGTTATAATTGGCTTTCCTGTGTAGTGATTATACATAGTTCCATATAGAGGGTCCGTCCCAACTAATATCATGTTAGTA

19F ST320 (3) *pbp2x* GCTGAGAAAATATAATTGGTAGAACCAACCAAGTATCCTCCATTTTTCTCATCAGCGATTTGAGCCGTACCGGATTTAACTGCTACATTTTGTCCAGGAACTGTTATAATTGGCTTTCCTGTGTAGTGATTATACATAGTTCCATATAGAGGGTCCGTCCCAACTAATATCATGTTAGTA

19A ST320 (1) *pbp2x* GCTGAGAAAATATAATTGGTAGAACCAACCAAGTATCCTCCATTTTTCTCATCAGCGATTTGAGCCGTACCGGATTTAACTGCTACATTTTGTCCAGGAACTGTTATAATTGGCTTTCCTGTGTAGTGATTATACATAGTTCCATATAGAGGGTCCGTCCCAACTAATATCATGTTAGTA

19A ST320 (2) *pbp2x* GCTGAGAAAATATAATTGGTAGAACCAACCAAGTATCCTCCATTTTTCTCATCAGCGATTTGAGCCGTACCGGATTTAACTGCTACATTTTGTCCAGGAACTGTTATAATTGGCTTTCCTGTGTAGTGATTATACATAGTTCCATATAGAGGGTCCGTCCCAACTAATATCATGTTAGTA

19A ST320 (3) *pbp2x* GCTGAGAAAATATAATTGGTAGAACCAACCAAGTATCCTCCATTTTTCTCATCAGCGATTTGAGCCGTACCGGATTTAACTGCTACATTTTGTCCAGGAACTGTTATAATTGGCTTTCCTGTGTAGTGATTATACATAGTTCCATATAGAGGGTCCGTCCCAACTAATATCATGTTAGTA

** ** ******** ** ** * ** ** * ** ** *********** ***** ** *********** ** ** * *********** ******** **** * ******* ****** *** ******** ******** * ** ******** ** ** * *********

19A ST199 (1) *pbp2x* CGAGTTAGACTAGCTGCATCTTTAGAAACAGGATTTCCCACAATTTCTTTTTGAGATTTCCGAGCAGTTTGATCATTTGGATCATAAATGGCACTAATAAATTTAGGCTCCAGCATGACACCGTCATTAGCAATAGCGGTAAAGGCACGAATCATTTGCGTCTGGGTCACTGAAATCCCT

19A ST199 (2) *pbp2x* CGAGTTAGACTAGCTGCATCTTTAGAAACAGGATTTCCCACAATTTCTTTTTGAGATTTCCGAGCAGTTTGATCATTTGGATCATAAATGGCACTAATAAATTTAGGCTCCAGCATGACACCGTCATTAGCAATAGCGGTAAAGGCACGAATCATTTGCGTCTGGGTCACTGAAATCCCT

19F ST320 (1) *pbp2x* CGAGTCAAGCTTGCTGTATCTTCAGATACAGGTTTTCCTACAATCTCTTTTTGAGACTTTCGTACAGACTGATTGTTAGTATCATAAATAGCACTTATAAATTTTGGCTCCAGCATAACTCCATCATTAGCAATAGCTGTAAAGGCACGAAGCATTTGTGTTTGTGTCACTGAAATTCCT

19F ST320 (2) *pbp2x* CGAGTCAAGCTTGCTGTATCTTCAGATACAGGTTTTCCTACAATCTCTTTTTGAGACTTTCGTACAGACTGATTGTTAGTATCATAAATAGCACTTATAAATTTTGGCTCCAGCATAACTCCATCATTAGCAATAGCTGTAAAGGCACGAAGCATTTGTGTTTGTGTCACTGAAATTCCT

19F ST320 (3) *pbp2x* CGAGTCAAGCTTGCTGTATCTTCAGATACAGGTTTTCCTACAATCTCTTTTTGAGACTTTCGTACAGACTGATTGTTAGTATCATAAATAGCACTTATAAATTTTGGCTCCAGCATAACTCCATCATTAGCAATAGCTGTAAAGGCACGAAGCATTTGTGTTTGTGTCACTGAAATTCCT

19A ST320 (1) *pbp2x* CGAGTCAAGCTTGCTGTATCTTCAGATACAGGTTTTCCTACAATCTCTTTTTGAGACTTTCGTACAGACTGATTGTTAGTATCATAAATAGCACTTATAAATTTTGGCTCCAGCATAACTCCATCATTAGCAATAGCTGTAAAGGCACGAAGCATTTGTGTTTGTGTCACTGAAATTCCT

19A ST320 (2) *pbp2x* CGAGTCAAGCTTGCTGTATCTTCAGATACAGGTTTTCCTACAATCTCTTTTTGAGACTTTCGTACAGACTGATTGTTAGTATCATAAATAGCACTTATAAATTTTGGCTCCAGCATAACTCCATCATTAGCAATAGCTGTAAAGGCACGAAGCATTTGTGTTTGTGTCACTGAAATTCCT

19A ST320 (3) *pbp2x* CGAGTCAAGCTTGCTGTATCTTCAGATACAGGTTTTCCTACAATCTCTTTTTGAGACTTTCGTACAGACTGATTGTTAGTATCATAAATAGCACTTATAAATTTTGGCTCCAGCATAACTCCATCATTAGCAATAGCTGTAAAGGCACGAAGCATTTGTGTTTGTGTCACTGAAATTCCT

***** * ** **** ***** *** ***** ***** ***** *********** ** ** *** **** ** * ********* ***** ******** *********** ** ** ************** ************* ****** ** ** *********** ***

19A ST199 (1) *pbp2x* TGTCCAAATGAGCTTTGCGCAATGTTGACAATATTATCCGCAGGAAGCTGACCAGCATACTCATCCGTCAAACCGAAACGGGTCGGAACACCAAATTTAAAGCGATTAAGATAATCAAGCCAGGTAGCATCTCCCATCTTTTGCTCAAGGAGGGTCATCCCAACGTTACTTGAGTGAGCA

19A ST199 (2) *pbp2x* TGTCCAAATGAGCTTTGCGCAATGTTGACAATATTATCCGCAGGAAGCTGACCAGCATACTCATCCGTCAAACCGAAACGGGTCGGAACACCAAATTTAAAGCGATTAAGATAATCAAGCCAGGTAGCATCTCCCATCTTTTGCTCAAGGAGGGTCATCCCAACGTTACTTGAGTGAGCA

19F ST320 (1) *pbp2x* TGCCCAAATGAGCTTTGAGCAATACTAACAATATTATCAGCTGGAAGTTGACCAGCGTATTCATCTGTCAAGCCAAAGCGAGTTGGAACCCCAAATTTAAAGCGTTTTAGATAATCCAACCAAGTAGCATCTCCCATTTTTTGTTCAAGTAGACTCATTCCAACATTACTGGAGTGAGCG

19F ST320 (2) *pbp2x* TGCCCAAATGAGCTTTGAGCAATACTAACAATATTATCAGCTGGAAGTTGACCAGCGTATTCATCTGTCAAGCCAAAGCGAGTTGGAACCCCAAATTTAAAGCGTTTTAGATAATCCAACCAAGTAGCATCTCCCATTTTTTGTTCAAGTAGACTCATTCCAACATTACTGGAGTGAGCG

19F ST320 (3) *pbp2x* TGCCCAAATGAGCTTTGAGCAATACTAACAATATTATCAGCTGGAAGTTGACCAGCGTATTCATCTGTCAAGCCAAAGCGAGTTGGAACCCCAAATTTAAAGCGTTTTAGATAATCCAACCAAGTAGCATCTCCCATTTTTTGTTCAAGTAGACTCATTCCAACATTACTGGAGTGAGCG

19A ST320 (1) *pbp2x* TGCCCAAATGAGCTTTGAGCAATACTAACAATATTATCAGCTGGAAGTTGACCAGCGTATTCATCTGTCAAGCCAAAGCGAGTTGGAACCCCAAATTTAAAGCGTTTTAGATAATCCAACCAAGTAGCATCTCCCATTTTTTGTTCAAGTAGACTCATTCCAACATTACTGGAGTGAGCG

19A ST320 (2) *pbp2x* TGCCCAAATGAGCTTTGAGCAATACTAACAATATTATCAGCTGGAAGTTGACCAGCGTATTCATCTGTCAAGCCAAAGCGAGTTGGAACCCCAAATTTAAAGCGTTTTAGATAATCCAACCAAGTAGCATCTCCCATTTTTTGTTCAAGTAGACTCATTCCAACATTACTGGAGTGAGCG

19A ST320 (3) *pbp2x* TGCCCAAATGAGCTTTGAGCAATACTAACAATATTATCAGCTGGAAGTTGACCAGCGTATTCATCTGTCAAGCCAAAGCGAGTTGGAACCCCAAATTTAAAGCGTTTTAGATAATCCAACCAAGTAGCATCTCCCATTTTTTGTTCAAGTAGACTCATTCCAACATTACTGGAGTGAGCG

** ************** ***** * *********** ** ***** ******** ** ***** ***** ** ** ** ** ***** ************** ** ******** * *** ************** ***** ***** ** **** ***** ***** ********

19A ST199 (1) *pbp2x* AATCCTTGAGAAAAGGTCATCATACCACCGCCGGTCAAACCTTCATTAACGTCCCAATCTCGGATCGTCACATCCGCTATTTTTAATTCACTGCTATTGAAATATTCTCCACCTGGGAAGGTATTGTTATCAATAGAAGCAGCTAACGTCATAACCTTCATGGCTGACCCTGGTTCATAG

19A ST199 (2) *pbp2x* AATCCTTGAGAAAAGGTCATCATACCACCGCCGGTCAAACCTTCATTAACGTCCCAATCTCGGATCGTCACATCCGCTATTTTTAATTCACTGCTATTGAAATATTCTCCACCTGGGAAGGTATTGTTATCAATAGAAGCAGCTAACGTCATAACCTTCATGGCTGACCCTGGTTCATAG

19F ST320 (1) *pbp2x* AAACCTTGTAAGAAAGTCATCATCCCACCAGTAGTCAAACCCTCATTAACATCCCAATCTCGAGTCGTCGCATCCGCTATTTTAAATTCACTGCTATTGAAGTATTCTCCACTTGGGAAGGTATTATTATCAATAGAAGAAGCTAACGTCATAACCTTCATGGCTGATCCTGGTTCATAG

19F ST320 (2) *pbp2x* AAACCTTGTAAGAAAGTCATCATCCCACCAGTAGTCAAACCCTCATTAACATCCCAATCTCGAGTCGTCGCATCCGCTATTTTAAATTCACTGCTATTGAAGTATTCTCCACTTGGGAAGGTATTATTATCAATAGAAGAAGCTAACGTCATAACCTTCATGGCTGATCCTGGTTCATAG

19F ST320 (3) *pbp2x* AAACCTTGTAAGAAAGTCATCATCCCACCAGTAGTCAAACCCTCATTAACATCCCAATCTCGAGTCGTCGCATCCGCTATTTTAAATTCACTGCTATTGAAGTATTCTCCACTTGGGAAGGTATTATTATCAATAGAAGAAGCTAACGTCATAACCTTCATGGCTGATCCTGGTTCATAG

19A ST320 (1) *pbp2x* AAACCTTGTAAGAAAGTCATCATCCCACCAGTAGTCAAACCCTCATTAACATCCCAATCTCGAGTCGTCGCATCCGCTATTTTAAATTCACTGCTATTGAAGTATTCTCCACTTGGGAAGGTATTATTATCAATAGAAGAAGCTAACGTCATAACCTTCATGGCTGATCCTGGTTCATAG

19A ST320 (2) *pbp2x* AAACCTTGTAAGAAAGTCATCATCCCACCAGTAGTCAAACCCTCATTAACATCCCAATCTCGAGTCGTCGCATCCGCTATTTTAAATTCACTGCTATTGAAGTATTCTCCACTTGGGAAGGTATTATTATCAATAGAAGAAGCTAACGTCATAACCTTCATGGCTGATCCTGGTTCATAG

19A ST320 (3) *pbp2x* AAACCTTGTAAGAAAGTCATCATCCCACCAGTAGTCAAACCCTCATTAACATCCCAATCTCGAGTCGTCGCATCCGCTATTTTAAATTCACTGCTATTGAAGTATTCTCCACTTGGGAAGGTATTATTATCAATAGAAGAAGCTAACGTCATAACCTTCATGGCTGATCCTGGTTCATAG

** ***** * ** ******** ***** ******** ******** *********** ***** ************* ***************** ********** ************ ************* *************************** ************

19A ST199 (1) *pbp2x* TTACTTTGATAAAGGATATCACGCCAAACAAAGTCCTTAGTGATTCCTTCTTTAGTATCTGCATTAAAGGTCGGTCGTTGGGTGGTAGCAAGGATTTCACCAGTCTTTGCACTGACCAAGGTCGCGGTCATGTACTTACCTTTTACCTTCTCTTGAAAAGCATCCATCTGGGTTTCCATA

19A ST199 (2) *pbp2x* TTACTTTGATAAAGGATATCACGCCAAACAAAGTCCTTAGTGATTCCTTCTTTAGTATCTGCATTAAAGGTCGGTCGTTGGGTGGTAGCAAGGATTTCACCAGTCTTTGCACTGACCAAGGTCGCGGTCATGTACTTACCTTTTACCTTCTCTTGAAAAGCATCCATCTGGGTTTCCATA

19F ST320 (1) *pbp2x* TTACTTTGATAAAGAATATCACGCCAAACAAAGTCCTCAGTGATTCCTTCTTTAGTATCTGCATTAAAGGTAGGTCGTTGGGTGGTAGCGAGGATTTCACCGGTCTTTGCACTGACCAAGGTCGCGGTCATATACTTACCTTTTACCTTTTCTAGAAAGGCATCCATCTGAGTTTCCATG

19F ST320 (2) *pbp2x* TTACTTTGATAAAGAATATCACGCCAAACAAAGTCCTCAGTGATTCCTTCTTTAGTATCTGCATTAAAGGTAGGTCGTTGGGTGGTAGCGAGGATTTCACCGGTCTTTGCACTGACCAAGGTCGCGGTCATATACTTACCTTTTACCTTTTCTAGAAAGGCATCCATCTGAGTTTCCATG

19F ST320 (3) *pbp2x* TTACTTTGATAAAGAATATCACGCCAAACAAAGTCCTCAGTGATTCCTTCTTTAGTATCTGCATTAAAGGTAGGTCGTTGGGTGGTAGCGAGGATTTCACCGGTCTTTGCACTGACCAAGGTCGCGGTCATATACTTACCTTTTACCTTTTCTAGAAAGGCATCCATCTGAGTTTCCATG

19A ST320 (1) *pbp2x* TTACTTTGATAAAGAATATCACGCCAAACAAAGTCCTCAGTGATTCCTTCTTTAGTATCTGCATTAAAGGTAGGTCGTTGGGTGGTAGCGAGGATTTCACCGGTCTTTGCACTGACCAAGGTCGCGGTCATATACTTACCTTTTACCTTTTCTAGAAAGGCATCCATCTGAGTTTCCATG

19A ST320 (2) *pbp2x* TTACTTTGATAAAGAATATCACGCCAAACAAAGTCCTCAGTGATTCCTTCTTTAGTATCTGCATTAAAGGTAGGTCGTTGGGTGGTAGCGAGGATTTCACCGGTCTTTGCACTGACCAAGGTCGCGGTCATATACTTACCTTTTACCTTTTCTAGAAAGGCATCCATCTGAGTTTCCATG

19A ST320 (3) *pbp2x* TTACTTTGATAAAGAATATCACGCCAAACAAAGTCCTCAGTGATTCCTTCTTTAGTATCTGCATTAAAGGTAGGTCGTTGGGTGGTAGCGAGGATTTCACCGGTCTTTGCACTGACCAAGGTCGCGGTCATATACTTACCTTTTACCTTTTCTAGAAAGGCATCCATCTGAGTTTCCATG

************** ********************** ********************************* ***************** *********** ***************************** ***************** *** **** *********** ********

19A ST199 (1) *pbp2x* AAGGACTGGAGGGGGCTGGAAATGGTTGTGTAAACATCCTTACCATCCACCGTTTGTTGGGAAATTTGTTCTGTTCCGGGGACAATATTTCCCAGACGATCCTTTTCATAGGTAATAATGCCGTCTGTCCCTGCAAGAATACTGTTCAAGGAACTCTCCATTCCAGAGGTTCCCAGCAAG

19A ST199 (2) *pbp2x* AAGGACTGGAGGGGGCTGGAAATGGTTGTGTAAACATCCTTACCATCCACCGTTTGTTGGGAAATTTGTTCTGTTCCGGGGACAATATTTCCCAGACGATCCTTTTCATAGGTAATAATGCCGTCTGTCCCTGCAAGAATACTGTTCAAGGAACTCTCCATTCCAGAGGTTCCCAGCAAG

19F ST320 (1) *pbp2x* AAAGATTGTAGCGGACTAGACAATGTTGTATAAACATCCTTGCCATCCACAGTTTGTTGCGATACCAGTTCTGTACCTGGTACGATATTTCCTACACGGTCTTTTTCATAGGTAATAATACCGTCTGTCCCTGCAAGAATACTGTTCAAGGAACTCTCCATTCCAGAGGTTCCCAGCAAA

19F ST320 (2) *pbp2x* AAAGATTGTAGCGGACTAGACAATGTTGTATAAACATCCTTGCCATCCACAGTTTGTTGCGATACCAGTTCTGTACCTGGTACGATATTTCCTACACGGTCTTTTTCATAGGTAATAATACCGTCTGTCCCTGCAAGAATACTGTTCAAGGAACTCTCCATTCCAGAGGTTCCCAGCAAA

19F ST320 (3) *pbp2x* AAAGATTGTAGCGGACTAGACAATGTTGTATAAACATCCTTGCCATCCACAGTTTGTTGCGATACCAGTTCTGTACCTGGTACGATATTTCCTACACGGTCTTTTTCATAGGTAATAATACCGTCTGTCCCTGCAAGAATACTGTTCAAGGAACTCTCCATTCCAGAGGTTCCCAGCAAA

19A ST320 (1) *pbp2x* AAAGATTGTAGCGGACTAGACAATGTTGTATAAACATCCTTGCCATCCACAGTTTGTTGCGATACCAGTTCTGTACCTGGTACGATATTTCCTACACGGTCTTTTTCATAGGTAATAATACCGTCTGTCCCTGCAAGAATACTGTTCAAGGAACTCTCCATTCCAGAGGTTCCCAGCAAA

19A ST320 (2) *pbp2x* AAAGATTGTAGCGGACTAGACAATGTTGTATAAACATCCTTGCCATCCACAGTTTGTTGCGATACCAGTTCTGTACCTGGTACGATATTTCCTACACGGTCTTTTTCATAGGTAATAATACCGTCTGTCCCTGCAAGAATACTGTTCAAGGAACTCTCCATTCCAGAGGTTCCCAGCAAA

19A ST320 (3) *pbp2x* AAAGATTGTAGCGGACTAGACAATGTTGTATAAACATCCTTGCCATCCACAGTTTGTTGCGATACCAGTTCTGTACCTGGTACGATATTTCCTACACGGTCTTTTTCATAGGTAATAATACCGTCTGTCCCTGCAAGAATACTGTTCAAGGAACTCTCCATTCCAGAGGTTCCCAGCAAA

** ** ** ** ** ** ** * ***** *********** ******** ******** ** * ******* ** ** ** ******** * *** ** ***************** ***********************************************************

19A ST199 (1) *pbp2x* CTCTTGCTGCCATCTTCATTTTCATGGAGCTGAGCTAGTCCGATAAAACTAGAAGCAAATTGTCCGTTTGGGTAACTACGATTGGGACTGGTTGTAAAATCAATCCCCTTGACCTCTGCAGTTTCCAACTCTTTTTTGATAGACATCATATTGGCATAGGTAATCCCATTGCCCTTTGCT

19A ST199 (2) *pbp2x* CTCTTGCTGCCATCTTCATTTTCATGGAGCTGAGCTAGTCCGATAAAACTAGAAGCAAATTGTCCGTTTGGGTAACTACGATTGGGACTGGTTGTAAAATCAATCCCCTTGACCTCTGCAGTTTCCAACTCTTTTTTGATAGACATCATATTGGCATAGGTAATCCCATTGCCCTTTGCT

19F ST320 (1) *pbp2x* CTCTTGCTTCCATCTTCATTTTCATGGAGCTGAGCTAGACCGATAAAACTAGAAGCAAATTGTCCGTTTGGGTAACTACGATTAGGACTGGTTGTAAAATCAATCCCCTTGACCTCTGCAGTTTCCAATTCTTTTTTGATAGACATCATATTGGCATAGGTAATCCCATTGCCCTTCGAA

19F ST320 (2) *pbp2x* CTCTTGCTTCCATCTTCATTTTCATGGAGCTGAGCTAGACCGATAAAACTAGAAGCAAATTGTCCGTTTGGGTAACTACGATTAGGACTGGTTGTAAAATCAATCCCCTTGACCTCTGCAGTTTCCAATTCTTTTTTGATAGACATCATATTGGCATAGGTAATCCCATTGCCCTTCGAA

19F ST320 (3) *pbp2x* CTCTTGCTTCCATCTTCATTTTCATGGAGCTGAGCTAGACCGATAAAACTAGAAGCAAATTGTCCGTTTGGGTAACTACGATTAGGACTGGTTGTAAAATCAATCCCCTTGACCTCTGCAGTTTCCAATTCTTTTTTGATAGACATCATATTGGCATAGGTAATCCCATTGCCCTTCGAA

19A ST320 (1) *pbp2x* CTCTTGCTTCCATCTTCATTTTCATGGAGCTGAGCTAGTCCGATAAAACTAGAAGCAAATTGTCCGTTTGGGTAACTACGATTGGGACTGGTTGTAAAATCAATCCCCTTGACCTCTGCAGTTTCCAACTCTTTTTTGATAGACATCATATTGGCATAGGTAATCCCATTGCCCTTCGAA

19A ST320 (2) *pbp2x* CTCTTGCTTCCATCTTCATTTTCATGGAGCTGAGCTAGTCCGATAAAACTAGAAGCAAATTGTCCGTTTGGGTAACTACGATTGGGACTGGTTGTAAAATCAATCCCCTTGACCTCTGCAGTTTCCAACTCTTTTTTGATAGACATCATATTGGCATAGGTAATCCCATTGCCCTTCGAA

19A ST320 (3) *pbp2x* CTCTTGCTTCCATCTTCATTTTCATGGAGCTGAGCTAGTCCGATAAAACTAGAAGCAAATTGTCCGTTTGGGTAACTACGATTGGGACTGGTTGTAAAATCAATCCCCTTGACCTCTGCAGTTTCCAACTCTTTTTTGATAGACATCATATTGGCATAGGTAATCCCATTGCCCTTCGAA

******** ***************************** ******************************************** ******************************************** *********************************************** *

19A ST199 (1) *pbp2x* CCAAAGGAAACTTGCTTGAGATTAGGTTGCGAGAGTTGCTCTCTTACATAGGATTCTTCCATGTCCAGATACTTATGAAAGACCTCTGCAACCTTGTTAAATTGTGTTTTTTCTACGTAAAGAATCTTACCCGTTGCTGACTTATAGTTCTCATCAATGACCGCATAGACATTATAAGAT

19A ST199 (2) *pbp2x* CCAAAGGAAACTTGCTTGAGATTAGGTTGCGAGAGTTGCTCTCTTACATAGGATTCTTCCATGTCCAGATACTTATGAAAGACCTCTGCAACCTTGTTAAATTGTGTTTTTTCTACGTAAAGAATCTTACCCGTTGCTGACTTATAGTTCTCATCAATGACCGCATAGACATTATAAGAT

19F ST320 (1) *pbp2x* CCAAAGGAAACTTGCTTGAGATTAGGTTGCGAGAGTTGCTCTCTTACATAGGATTCTTCCATGTCCAGATACTTATGAAAGACCTCTGCAACCTTGTTAAATTGTGTTTTTTCTACGTAAAGAATCTTACCCGTTGCTGACTTATAGTTCTCATCAATGACCGCATAGACATTATAGGAG

19F ST320 (2) *pbp2x* CCAAAGGAAACTTGCTTGAGATTAGGTTGCGAGAGTTGCTCTCTTACATAGGATTCTTCCATGTCCAGATACTTATGAAAGACCTCTGCAACCTTGTTAAATTGTGTTTTTTCTACGTAAAGAATCTTACCCGTTGCTGACTTATAGTTCTCATCAATGACCGCATAGACATTATAGGAG

19F ST320 (3) *pbp2x* CCAAAGGAAACTTGCTTGAGATTAGGTTGCGAGAGTTGCTCTCTTACATAGGATTCTTCCATGTCCAGATACTTATGAAAGACCTCTGCAACCTTGTTAAATTGTGTTTTTTCTACGTAAAGAATCTTACCCGTTGCTGACTTATAGTTCTCATCAATGACCGCATAGACATTATAGGAG

19A ST320 (1) *pbp2x* CCAAAGGAAACTTGCTTGAGATTAGGTTGCGAGAGTTGCTCTCTTACATAGGATTCTTCCATGTCCAGATACTTATGAAAGACCTCTGCAACCTTGTTAAATTGTGTTTTTTCTACGTAAAGAATCTTACCCGTTGCTGACTTATAGTTCTCATCAATGACCGCATAGACATTATAGGAG

19A ST320 (2) *pbp2x* CCAAAGGAAACTTGCTTGAGATTAGGTTGCGAGAGTTGCTCTCTTACATAGGATTCTTCCATGTCCAGATACTTATGAAAGACCTCTGCAACCTTGTTAAATTGTGTTTTTTCTACGTAAAGAATCTTACCCGTTGCTGACTTATAGTTCTCATCAATGACCGCATAGACATTATAGGAG

19A ST320 (3) *pbp2x* CCAAAGGAAACTTGCTTGAGATTAGGTTGCGAGAGTTGCTCTCTTACATAGGATTCTTCCATGTCCAGATACTTATGAAAGACCTCTGCAACCTTGTTAAATTGTGTTTTTTCTACGTAAAGAATCTTACCCGTTGCTGACTTATAGTTCTCATCAATGACCGCATAGACATTATAGGAG

******************************************************************************************************************************************************************************** **

19A ST199 (1) *pbp2x* GTCGCATCTTCAGCAATCGGGACTCCATTTCGGTCATAAATAGTCCCACGTTTGGCAGGAACTGTACGGGTGGTTTGATGAACCTTCTTAGCTTCCTTCGCTAAATCTGTTCCAAAGCGAGTGCCCGTCCCAATAATGACCGCAAAATTGACTAAAAAAACGGCAAAAACAAAGACAGAT

19A ST199 (2) *pbp2x* GTCGCATCTTCAGCAATCGGGACTCCATTTCGGTCATAAATAGTCCCACGTTTGGCAGGAACTGTACGGGTGGTTTGATGAACCTTCTTAGCTTCCTTCGCTAAATCTGTTCCAAAGCGAGTGCCCGTCCCAATAATGACCGCAAAATTGACTAAAAAAACGGCAAAAACAAAGACAGAT

19F ST320 (1) *pbp2x* GTTGCATCCTCAGCAATCGGGACTCCATTTCGGTCATAAATAGTCCCACGTTTGGCAGGAACTGTACGGGTGGTTTGATGAACCTTCTTAGCTTCCTTCGCTAAATCTGTTCCAAAGCGAGTGCCTGTCCCAATAATGACCGCAAAATTGACTAAAAAAATGGCAAAAACAAAGACAGAT

19F ST320 (2) *pbp2x* GTTGCATCCTCAGCAATCGGGACTCCATTTCGGTCATAAATAGTCCCACGTTTGGCAGGAACTGTACGGGTGGTTTGATGAACCTTCTTAGCTTCCTTCGCTAAATCTGTTCCAAAGCGAGTGCCTGTCCCAATAATGACCGCAAAATTGACTAAAAAAATGGCAAAAACAAAGACAGAT

19F ST320 (3) *pbp2x* GTTGCATCCTCAGCAATCGGGACTCCATTTCGGTCATAAATAGTCCCACGTTTGGCAGGAACTGTACGGGTGGTTTGATGAACCTTCTTAGCTTCCTTCGCTAAATCTGTTCCAAAGCGAGTGCCTGTCCCAATAATGACCGCAAAATTGACTAAAAAAATGGCAAAAACAAAGACAGAT

19A ST320 (1) *pbp2x* GTTGCATCCTCAGCAATCGGGACTCCATTTCGGTCATAAATAGTCCCACGTTTGGCAGGAACTGTACGGGTGGTTTGATGAACCTTCTTAGCTTCCTTCGCTAAATCTGTTCCAAAGCGAGTGCCCGTCCCAATAATGACCGCAAAATTGACTAAAAAAACGGCAAAAACAAAGACAGAT

19A ST320 (2) *pbp2x* GTTGCATCCTCAGCAATCGGGACTCCATTTCGGTCATAAATAGTCCCACGTTTGGCAGGAACTGTACGGGTGGTTTGATGAACCTTCTTAGCTTCCTTCGCTAAATCTGTTCCAAAGCGAGTGCCCGTCCCAATAATGACCGCAAAATTGACTAAAAAAACGGCAAAAACAAAGACAGAT

19A ST320 (3) *pbp2x* GTTGCATCCTCAGCAATCGGGACTCCATTTCGGTCATAAATAGTCCCACGTTTGGCAGGAACTGTACGGGTGGTTTGATGAACCTTCTTAGCTTCCTTCGCTAAATCTGTTCCAAAGCGAGTGCCCGTCCCAATAATGACCGCAAAATTGACTAAAAAAACGGCAAAAACAAAGACAGAT

** ***** ******************************************************************************************************************** ********************************** *******************

19A ST199 (1) *pbp2x* AATAAACTCAGACTTTTTCCAACTCTGCGTCTGTTTTCAGCCGGCGATTTCCGATTTTTCGTCGCATAACGGATTACTCTTTTTGTCCACTTC

19A ST199 (2) *pbp2x* AATAAACTCAGACTTTTTCCAACTCTGCGTCTGTTTTCAGCCGGCGATTTCCGATTTTTCGTCGCATAACGGATTACTCTTTTTGTCCACTTC

19F ST320 (1) *pbp2x* AATAAACTCAGACTTTTTCCAACTCTGCGTCTGTTTTCAGCCGGCGATTTCCGATTTTTGGTCGCATAACGGATTACTCTTTTTGTCCACTTC

19F ST320 (2) *pbp2x* AATAAACTCAGACTTTTTCCAACTCTGCGTCTGTTTTCAGCCGGCGATTTCCGATTTTTGGTCGCATAACGGATTACTCTTTTTGTCCACTTC

19F ST320 (3) *pbp2x* AATAAACTCAGACTTTTTCCAACTCTGCGTCTGTTTTCAGCCGGCGATTTCCGATTTTTGGTCGCATAACGGATTACTCTTTTTGTCCACTTC

19A ST320 (1) *pbp2x* AATAAACTCAGACTTTTTCCAACTCTGCGTCTGTTTTCAGCCGGCGATTTCCGATTTTTGGTCGCATAACGGATTACTTTTTTTGTCCACTTC

19A ST320 (2) *pbp2x* AATAAACTCAGACTTTTTCCAACTCTGCGTCTGTTTTCAGCCGGCGATTTCCGATTTTTGGTCGCATAACGGATTACTTTTTTTGTCCACTTC

19A ST320 (3) *pbp2x* AATAAACTCAGACTTTTTCCAACTCTGCGTCTGTTTTCAGCCGGCGATTTCCGATTTTTGGTCGCATAACGGATTACTTTTTTTGTCCACTTC

*********************************************************** ****************** **************

MUSCLE (3.7) multiple sequence alignment

19A ST199 (1) *pbp1a* ATGAACAAACCAACGATTCTGCGCCTAATCAAGTATCTGAGCATTAGCTTCTTAAGCTTGGTTATCGCAGCCATTGTCTTAGGCGGAGGAGTTTTTTTCTACTACGTTAGCAAGGCTCCTAGCCTATCCGAGAGTAAACTAGTTGCAACAACCTCTAGTAAAATCTACGACAATAAAAAT

19A ST199 (2) *pbp1a* ATGAACAAACCAACGATTCTGCGCCTAATCAAGTATCTGAGCATTAGCTTCTTAAGCTTGGTTATCGCAGCCATTGTCTTAGGCGGAGGAGTTTTTTTCTACTACGTTAGCAAGGCTCCTAGCCTATCCGAGAGTAAACTAGTTGCAACAACCTCTAGTAAAATCTACGACAATAAAAAT

19F ST320 (1) *pbp1a* ATGAACAAACAAACTATCCTGCGAATAGCTAAGTATGTGAGTATCTGCTTCTTAACTTTATTTATCACAGCAGTTATGCTGGGAGGAGGCATATTTCTCTATTACGTCAGCAAAGCCCCAGCCCTATCTGAGAGCAAATTAGTCGCAACCACGTCTAGTAAGATTTTCGATAGTAAAAAC

19F ST320 (2) *pbp1a* ATGAACAAACAAACTATCCTGCGAATAGCTAAGTATGTGAGTATCTGCTTCTTAACTTTATTTATCACAGCAGTTATGCTGGGAGGAGGCATATTTCTCTATTACGTCAGCAAAGCCCCAGCCCTATCTGAGAGCAAATTAGTCGCAACCACGTCTAGTAAGATTTTCGATAGTAAAAAC

19F ST320 (3) *pbp1a* ATGAACAAACAAACTATCCTGCGAATAGCTAAGTATGTGAGTATCTGCTTCTTAACTTTATTTATCACAGCAGTTATGCTGGGAGGAGGCATATTTCTCTATTACGTCAGCAAAGCCCCAGCCCTATCTGAGAGCAAATTAGTCGCAACCACGTCTAGTAAGATTTTCGATAGTAAAAAC

19A ST320 (1) *pbp1a* ATGAACAAACAAACTATCCTGCGAATAGCTAAGTATGTGAGTATCTGCTTCTTAACTTTATTTATCACAGCAGTTATGCTGGGAGGAGGCATATTTCTCTATTACGTCAGCAAAGCCCCAGCCCTATCTGAGAGCAAATTAGTCGCAACCACGTCTAGTAAGATTTTCGATAGTAAAAAC

19A ST320 (2) *pbp1a* ATGAACAAACAAACTATCCTGCGAATAGCTAAGTATGTGAGTATCTGCTTCTTAACTTTATTTATCACAGCAGTTATGCTGGGAGGAGGCATATTTCTCTATTACGTCAGCAAAGCCCCAGCCCTATCTGAGAGCAAATTAGTCGCAACCACGTCTAGTAAGATTTTCGATAGTAAAAAC

19A ST320 (3) *pbp1a* ATGAACAAACAAACTATCCTGCGAATAGCTAAGTATGTGAGTATCTGCTTCTTAACTTTATTTATCACAGCAGTTATGCTGGGAGGAGGCATATTTCTCTATTACGTCAGCAAAGCCCCAGCCCTATCTGAGAGCAAATTAGTCGCAACCACGTCTAGTAAGATTTTCGATAGTAAAAAC

********** *** ** ***** ** ****** **** ** ********* ** ***** **** ** * * ** ***** * *** **** ***** ***** ** ** ****** ***** *** **** ***** ** ******** ** * *** * ******

19A ST199 (1) *pbp1a* CAACTCATTGCTGACTTGGGTTCTGAACGCCGCGTCAATGCCCAAGCTAATGATATTCCCACAGATTTGGTTAAGGCAATCGTTTCTATCGAAGACCATCGCTTCTTCGACCACAGGGGGATTGATACCATCCGTATCCTGGGAGCTTTCTTGCGCAATCTGCAAAGCAATTCCCTCCAA

19A ST199 (2) *pbp1a* CAACTCATTGCTGACTTGGGTTCTGAACGCCGCGTCAATGCCCAAGCTAATGATATTCCCACAGATTTGGTTAAGGCAATCGTTTCTATCGAAGACCATCGCTTCTTCGACCACAGGGGGATTGATACCATCCGTATCCTGGGAGCTTTCTTGCGCAATCTGCAAAGCAATTCCCTCCAA

19F ST320 (1) *pbp1a* GAACTGATTGCCGATCTGGGCTCTGAACGCCGAGTAAACGCTCAGGCCAACGAAATTCCTACTGATTTGGTCAAGGCTATCGTATCGATCGAAGACCATCGTTTTTTCGATCATCGCGGTGTCGATACCATCCGAATTATAGGAGCTTTTTTACGTAACTTACAAAACAACTCCCTCCAG

19F ST320 (2) *pbp1a* GAACTGATTGCCGATCTGGGCTCTGAACGCCGAGTAAACGCTCAGGCCAACGAAATTCCTACTGATTTGGTCAAGGCTATCGTATCGATCGAAGACCATCGTTTTTTCGATCATCGCGGTGTCGATACCATCCGAATTATAGGAGCTTTTTTACGTAACTTACAAAACAACTCCCTCCAG

19F ST320 (3) *pbp1a* GAACTGATTGCCGATCTGGGCTCTGAACGCCGAGTAAACGCTCAGGCCAACGAAATTCCTACTGATTTGGTCAAGGCTATCGTATCGATCGAAGACCATCGTTTTTTCGATCATCGCGGTGTCGATACCATCCGAATTATAGGAGCTTTTTTACGTAACTTACAAAACAACTCCCTCCAG

19A ST320 (1) *pbp1a* GAACTGATTGCCGATCTGGGCTCTGAACGCCGAGTAAACGCTCAGGCCAACGAAATTCCTACTGATTTGGTCAAGGCTATCGTATCGATCGAAGACCATCGTTTTTTCGATCATCGCGGTGTCGATACCATCCGAATTATAGGAGCTTTTTTACGTAACTTACAAAACAACTCCCTCCAG

19A ST320 (2) *pbp1a* GAACTGATTGCCGATCTGGGCTCTGAACGCCGAGTAAACGCTCAGGCCAACGAAATTCCTACTGATTTGGTCAAGGCTATCGTATCGATCGAAGACCATCGTTTTTTCGATCATCGCGGTGTCGATACCATCCGAATTATAGGAGCTTTTTTACGTAACTTACAAAACAACTCCCTCCAG

19A ST320 (3) *pbp1a* GAACTGATTGCCGATCTGGGCTCTGAACGCCGAGTAAACGCTCAGGCCAACGAAATTCCTACTGATTTGGTCAAGGCTATCGTATCGATCGAAGACCATCGTTTTTTCGATCATCGCGGTGTCGATACCATCCGAATTATAGGAGCTTTTTTACGTAACTTACAAAACAACTCCCTCCAG

**** ***** ** **** *********** ** ** ** ** ** ** ** ***** ** ******** ***** ***** ** ************** ** ***** ** * ** * *********** ** * ******** ** ** ** * **** *** ********

19A ST199 (1) *pbp1a* GGTGGATCAACTCTCACCCAACAGTTGATTAAGTTGACTTACTTTTCAACCTCGACTTCCGACCAGACTATTTCTCGTAAGGCTCAGGAAGCTTGGTTAGCGATTCAGTTAGAACAAAAAGCAACCAAGCAAGAAATCTTGACCTACTATATAAATAAGGTCTACATGTCTAATGGCAAC

19A ST199 (2) *pbp1a* GGTGGATCAACTCTCACCCAACAGTTGATTAAGTTGACTTACTTTTCAACCTCGACTTCCGACCAGACTATTTCTCGTAAGGCTCAGGAAGCTTGGTTAGCGATTCAGTTAGAACAAAAAGCAACCAAGCAAGAAATCTTGACCTACTATATAAATAAGGTCTACATGTCTAATGGCAAC

19F ST320 (1) *pbp1a* GGAGGATCTACCCTTACCCAACAATTAATTAAGTTGACCTATTTCTCAACTTCCACTTCTGATCAGACCATTTCACGTAAAGCCCAGGAAGCTTGGTTGGCTGTTCAATTAGAACAAAAAGCCACAAAACAAGAAATCCTGACCTACTATGTGAATAAAGTTTACATGTCTAACGGAAAC

19F ST320 (2) *pbp1a* GGAGGATCTACCCTTACCCAACAATTAATTAAGTTGACCTATTTCTCAACTTCCACTTCTGATCAGACCATTTCACGTAAAGCCCAGGAAGCTTGGTTGGCTGTTCAATTAGAACAAAAAGCCACAAAACAAGAAATCCTGACCTACTATGTGAATAAAGTTTACATGTCTAACGGAAAC

19F ST320 (3) *pbp1a* GGAGGATCTACCCTTACCCAACAATTAATTAAGTTGACCTATTTCTCAACTTCCACTTCTGATCAGACCATTTCACGTAAAGCCCAGGAAGCTTGGTTGGCTGTTCAATTAGAACAAAAAGCCACAAAACAAGAAATCCTGACCTACTATGTGAATAAAGTTTACATGTCTAACGGAAAC

19A ST320 (1) *pbp1a* GGAGGATCTACCCTTACCCAACAATTAATTAAGTTGACCTATTTCTCAACTTCCACTTCTGATCAGACCATTTCACGTAAAGCCCAGGAAGCTTGGTTGGCTGTTCAATTAGAACAAAAAGCCACAAAACAAGAAATCCTGACCTACTATGTGAATAAAGTTTACATGTCTAACGGAAAC

19A ST320 (2) *pbp1a* GGAGGATCTACCCTTACCCAACAATTAATTAAGTTGACCTATTTCTCAACTTCCACTTCTGATCAGACCATTTCACGTAAAGCCCAGGAAGCTTGGTTGGCTGTTCAATTAGAACAAAAAGCCACAAAACAAGAAATCCTGACCTACTATGTGAATAAAGTTTACATGTCTAACGGAAAC

19A ST320 (3) *pbp1a* GGAGGATCTACCCTTACCCAACAATTAATTAAGTTGACCTATTTCTCAACTTCCACTTCTGATCAGACCATTTCACGTAAAGCCCAGGAAGCTTGGTTGGCTGTTCAATTAGAACAAAAAGCCACAAAACAAGAAATCCTGACCTACTATGTGAATAAAGTTTACATGTCTAACGGAAAC

** ***** ** ** ******** ** *********** ** ** ***** ** ***** ** ***** ***** ***** ** ************** ** **** ************** ** ** ********* *********** * ***** ** *********** ** ***

19A ST199 (1) *pbp1a* TATGGAATGCAGACAGCAGCTCAAAACTACTATGGTAAAGACCTCAATAATTTAAGTTTACCTCAGTTAGCCTTGCTGGCTGGAATGCCTCAGGCACCAAACCAATATGACCCCTATTCACATCCAGAAGCAGCCCAAGACCGCCGAAACTTGGTCTTATCTGAAATGAAAAATCAAGGT

19A ST199 (2) *pbp1a* TATGGAATGCAGACAGCAGCTCAAAACTACTATGGTAAAGACCTCAATAATTTAAGTTTACCTCAGTTAGCCTTGCTGGCTGGAATGCCTCAGGCACCAAACCAATATGACCCCTATTCACATCCAGAAGCAGCCCAAGACCGCCGAAACTTGGTCTTATCTGAAATGAAAAATCAAGGT

19F ST320 (1) *pbp1a* TATGGAATGCAGACGGCTGCCCAAAACTATTACGGCAAAGATTTGCGAGAACTATCATTACCTCAACTTGCCCTACTAGCAGGAATGCCACAGGCTCCGAATCAATACGATCCCTATTCTCATCCAGAAGCTGCCCTTGACCGTCGTAACTTGGTACTTTCAGAGATGAAAGGTCAAGGC

19F ST320 (2) *pbp1a* TATGGAATGCAGACGGCTGCCCAAAACTATTACGGCAAAGATTTGCGAGAACTATCATTACCTCAACTTGCCCTACTAGCAGGAATGCCACAGGCTCCGAATCAATACGATCCCTATTCTCATCCAGAAGCTGCCCTTGACCGTCGTAACTTGGTACTTTCAGAGATGAAAGGTCAAGGC

19F ST320 (3) *pbp1a* TATGGAATGCAGACGGCTGCCCAAAACTATTACGGCAAAGATTTGCGAGAACTATCATTACCTCAACTTGCCCTACTAGCAGGAATGCCACAGGCTCCGAATCAATACGATCCCTATTCTCATCCAGAAGCTGCCCTTGACCGTCGTAACTTGGTACTTTCAGAGATGAAAGGTCAAGGC

19A ST320 (1) *pbp1a* TATGGAATGCAGACGGCTGCCCAAAACTATTACGGCAAAGATTTGCGAGAACTATCATTACCTCAACTTGCCCTACTAGCAGGAATGCCACAGGCTCCGAATCAATACGATCCCTATTCTCATCCAGAAGCTGCCCTTGACCGTCGTAACTTGGTACTTTCAGAGATGAAAGGTCAAGGC

19A ST320 (2) *pbp1a* TATGGAATGCAGACGGCTGCCCAAAACTATTACGGCAAAGATTTGCGAGAACTATCATTACCTCAACTTGCCCTACTAGCAGGAATGCCACAGGCTCCGAATCAATACGATCCCTATTCTCATCCAGAAGCTGCCCTTGACCGTCGTAACTTGGTACTTTCAGAGATGAAAGGTCAAGGC

19A ST320 (3) *pbp1a* TATGGAATGCAGACGGCTGCCCAAAACTATTACGGCAAAGATTTGCGAGAACTATCATTACCTCAACTTGCCCTACTAGCAGGAATGCCACAGGCTCCGAATCAATACGATCCCTATTCTCATCCAGAAGCTGCCCTTGACCGTCGTAACTTGGTACTTTCAGAGATGAAAGGTCAAGGC

************** ** ** ******** ** ** ***** * * ** ******** * *** * ** ** ******** ***** ** ** ***** ** ******** *********** **** ***** ** ******** * ** ** ****** ******

19A ST199 (1) *pbp1a* TACATCTCTGCTGAACAGTATGAGAAAGCAGTCAATACACCAATTACTGATGGACTACAAAGTCTCAAATCAGCAAGTAATTACCCTGCTTACATGGATAATTACCTCAAGGAAGTCATCAATGTAGACCAAGAAGCTCAAAAACATCTGTGGGATATTTACAATACAGACGAATACGTT

19A ST199 (2) *pbp1a* TACATCTCTGCTGAACAGTATGAGAAAGCAGTCAATACACCAATTACTGATGGACTACAAAGTCTCAAATCAGCAAGTAATTACCCTGCTTACATGGATAATTACCTCAAGGAAGTCATCAATGTAGACCAAGAAGCTCAAAAACATCTGTGGGATATTTACAATACAGACGAATACGTT

19F ST320 (1) *pbp1a* TACATTTCTGCCGAACAGTATGAGAAGGCTATTAATACCCCTATTACTGATGGACTCCAAAGTTTGAAATCGGTCAATAGCTATCCAGCATATATGGACAATTATCTCAAAGAGGTTATCAATGTAGACCAAGAAGCTCAAAAACATCTGTGGGATATCTACAACTCCGATCAATACGTC

19F ST320 (2) *pbp1a* TACATTTCTGCCGAACAGTATGAGAAGGCTATTAATACCCCTATTACTGATGGACTCCAAAGTTTGAAATCGGTCAATAGCTATCCAGCATATATGGACAATTATCTCAAAGAGGTTATCAATGTAGACCAAGAAGCTCAAAAACATCTGTGGGATATCTACAACTCCGATCAATACGTC

19F ST320 (3) *pbp1a* TACATTTCTGCCGAACAGTATGAGAAGGCTATTAATACCCCTATTACTGATGGACTCCAAAGTTTGAAATCGGTCAATAGCTATCCAGCATATATGGACAATTATCTCAAAGAGGTTATCAATGTAGACCAAGAAGCTCAAAAACATCTGTGGGATATCTACAACTCCGATCAATACGTC

19A ST320 (1) *pbp1a* TACATTTCTGCCGAACAGTATGAGAAGGCTATTAATACCCCTATTACTGATGGACTCCAAAGTTTGAAATCGGTCAATAGCTATCCAGCATATATGGACAATTATCTCAAAGAGGTTATCAATGTAGACCAAGAAGCTCAAAAACATCTGTGGGATATCTACAACTCCGATCAATACGTC

19A ST320 (2) *pbp1a* TACATTTCTGCCGAACAGTATGAGAAGGCTATTAATACCCCTATTACTGATGGACTCCAAAGTTTGAAATCGGTCAATAGCTATCCAGCATATATGGACAATTATCTCAAAGAGGTTATCAATGTAGACCAAGAAGCTCAAAAACATCTGTGGGATATCTACAACTCCGATCAATACGTC

19A ST320 (3) *pbp1a* TACATTTCTGCCGAACAGTATGAGAAGGCTATTAATACCCCTATTACTGATGGACTCCAAAGTTTGAAATCGGTCAATAGCTATCCAGCATATATGGACAATTATCTCAAAGAGGTTATCAATGTAGACCAAGAAGCTCAAAAACATCTGTGGGATATCTACAACTCCGATCAATACGTC

***** ***** ************** ** * ***** ** ************** ****** * ***** * * ** ** ** ** ** ***** ***** ***** ** ** ***************************************** ***** * ** *******

19A ST199 (1) *pbp1a* GCCTATCCAGACGATGAATTGCAAGTCGCTTCTACCATTGTTGATGTTTCTAACGGTAAAGTCATTGCCCAGCTAGGAGCACGCCATCAGTCAAGTAATGTTTCCTTCGGAATTAACCAAGCAGTGGAAACAAACCGCGACTGGGGATCAACTATGAAACCGATCACAGACTATGCTCCT

19A ST199 (2) *pbp1a* GCCTATCCAGACGATGAATTGCAAGTCGCTTCTACCATTGTTGATGTTTCTAACGGTAAAGTCATTGCCCAGCTAGGAGCACGCCATCAGTCAAGTAATGTTTCCTTCGGAATTAACCAAGCAGTGGAAACAAACCGCGACTGGGGATCAACTATGAAACCGATCACAGACTATGCTCCT

19F ST320 (1) *pbp1a* TCTTACCCTGACGATGATTTGCAAGTCGCATCTACGGTCGTAGATGTTTCAAATGGTAAAGTCATCGCACAACTTGGTGCTCGTCATCAAGCAAGTAATGTTTCATTCGGTACCAACCAGGCCGTAGAAACCAATCGTGACTGGGGATCATCAATGAAACCAATCACTGACTATGCTCCC

19F ST320 (2) *pbp1a* TCTTACCCTGACGATGATTTGCAAGTCGCATCTACGGTCGTAGATGTTTCAAATGGTAAAGTCATCGCACAACTTGGTGCTCGTCATCAAGCAAGTAATGTTTCATTCGGTACCAACCAGGCCGTAGAAACCAATCGTGACTGGGGATCATCAATGAAACCAATCACTGACTATGCTCCC

19F ST320 (3) *pbp1a* TCTTACCCTGACGATGATTTGCAAGTCGCATCTACGGTCGTAGATGTTTCAAATGGTAAAGTCATCGCACAACTTGGTGCTCGTCATCAAGCAAGTAATGTTTCATTCGGTACCAACCAGGCCGTAGAAACCAATCGTGACTGGGGATCATCAATGAAACCAATCACTGACTATGCTCCC

19A ST320 (1) *pbp1a* TCTTACCCTGACGATGATTTGCAAGTCGCATCTACGGTCGTAGATGTTTCAAATGGTAAAGTCATCGCACAACTTGGTGCTCGTCATCAAGCAAGTAATGTTTCATTCGGTACCAACCAGGCCGTAGAAACCAATCGTGACTGGGGATCATCAATGAAACCAATCACTGACTATGCTCCC

19A ST320 (2) *pbp1a* TCTTACCCTGACGATGATTTGCAAGTCGCATCTACGGTCGTAGATGTTTCAAATGGTAAAGTCATCGCACAACTTGGTGCTCGTCATCAAGCAAGTAATGTTTCATTCGGTACCAACCAGGCCGTAGAAACCAATCGTGACTGGGGATCATCAATGAAACCAATCACTGACTATGCTCCC

19A ST320 (3) *pbp1a* TCTTACCCTGACGATGATTTGCAAGTCGCATCTACGGTCGTAGATGTTTCAAATGGTAAAGTCATCGCACAACTTGGTGCTCGTCATCAAGCAAGTAATGTTTCATTCGGTACCAACCAGGCCGTAGAAACCAATCGTGACTGGGGATCATCAATGAAACCAATCACTGACTATGCTCCC

* ** ** ******** *********** ***** * ** ******** ** *********** ** ** ** ** ** ** ***** ************* ***** * ***** ** ** ***** ** ** ************ * ******** ***** ***********

19A ST199 (1) *pbp1a* GCCTTGGAATACGGTGTCTACGATTCAACTGCTACTATCGTTCACGATGAGCCCTATAACTATCCTGGGACAGATACCCCTGTTTATAACTGGGATAGGGGCTACTTTGGCAATATCACCTTGCAATACGCCCTGCAACAATCGCGAAACGTCCCAGCCGTGGAAACGCTAAACAAGGTC

19A ST199 (2) *pbp1a* GCCTTGGAATACGGTGTCTACGATTCAACTGCTACTATCGTTCACGATGAGCCCTATAACTATCCTGGGACAGATACCCCTGTTTATAACTGGGATAGGGGCTACTTTGGCAATATCACCTTGCAATACGCCCTGCAACAATCGCGAAACGTCCCAGCCGTGGAAACGCTAAACAAGGTC

19F ST320 (1) *pbp1a* GCTTTAGAATATGGAGTCTATGACTCTACTGCTTCTATTGTACATGATGTCCCTTATAACTATCCTGGCACTGATACTCCACTCTACAACTGGGATCATGTCTACTTTGGAAACATTACAATCCAGTATGCTCTTCAACAATCACGAAATGTCACAGCCGTTGAGACTTTGAATAAGGTC

19F ST320 (2) *pbp1a* GCTTTAGAATATGGAGTCTATGACTCTACTGCTTCTATTGTACATGATGTCCCTTATAACTATCCTGGCACTGATACTCCACTCTACAACTGGGATCATGTCTACTTTGGAAACATTACAATCCAGTATGCTCTTCAACAATCACGAAATGTCACAGCCGTTGAGACTTTGAATAAGGTC

19F ST320 (3) *pbp1a* GCTTTAGAATATGGAGTCTATGACTCTACTGCTTCTATTGTACATGATGTCCCTTATAACTATCCTGGCACTGATACTCCACTCTACAACTGGGATCATGTCTACTTTGGAAACATTACAATCCAGTATGCTCTTCAACAATCACGAAATGTCACAGCCGTTGAGACTTTGAATAAGGTC

19A ST320 (1) *pbp1a* GCTTTAGAATATGGAGTCTATGACTCTACTGCTTCTATTGTACATGATGTCCCTTATAACTATCCTGGCACTGATACTCCACTCTACAACTGGGATCATGTCTACTTTGGAAACATTACAATCCAGTATGCTCTTCAACAATCACGAAATGTCACAGCCGTTGAGACTTTGAATAAGGTC

19A ST320 (2) *pbp1a* GCTTTAGAATATGGAGTCTATGACTCTACTGCTTCTATTGTACATGATGTCCCTTATAACTATCCTGGCACTGATACTCCACTCTACAACTGGGATCATGTCTACTTTGGAAACATTACAATCCAGTATGCTCTTCAACAATCACGAAATGTCACAGCCGTTGAGACTTTGAATAAGGTC

19A ST320 (3) *pbp1a* GCTTTAGAATATGGAGTCTATGACTCTACTGCTTCTATTGTACATGATGTCCCTTATAACTATCCTGGCACTGATACTCCACTCTACAACTGGGATCATGTCTACTTTGGAAACATTACAATCCAGTATGCTCTTCAACAATCACGAAATGTCACAGCCGTTGAGACTTTGAATAAGGTC

** ** ***** ** ***** ** ** ****** **** ** ** **** ** ************** ** ***** ** * ** ********* * ********* ** ** ** * ** ** ** ** ******** ***** *** ******* ** ** * ** ******

19A ST199 (1) *pbp1a* GGACTCAACCGTGCCAAGACTTTCCTAAATGGTCTCGGAATCGACTACCCAAGTCTTCACTACTCAAATGCCATTTCAAGTAACACAACCGAGTCAGACAAAAAATATGGAGCAAGTAGTGAAAAGATGGCTGCTGCTTACGCTGCCTTTGCAAATGGTGGAACTTACTATAAACCAATG

19A ST199 (2) *pbp1a* GGACTCAACCGTGCCAAGACTTTCCTAAATGGTCTCGGAATCGACTACCCAAGTCTTCACTACTCAAATGCCATTTCAAGTAACACAACCGAGTCAGACAAAAAATATGGAGCAAGTAGTGAAAAGATGGCTGCTGCTTACGCTGCCTTTGCAAATGGTGGAACTTACTATAAACCAATG

19F ST320 (1) *pbp1a* GGTCTAGATAGAGCTAAAACCTTCCTTAATGGTCTTGGTATCGACTATCCAAGCATGCATTATGCAAACGCCATTTCAAGTAACACAACTGAATCCAACAAAAAATATGGTGCAAGTAGTGAAAAAATGGCTGCTGCCTACGCTGCTTTTGCTAATGGTGGTATTTATCACAAACCAATG

19F ST320 (2) *pbp1a* GGTCTAGATAGAGCTAAAACCTTCCTTAATGGTCTTGGTATCGACTATCCAAGCATGCATTATGCAAACGCCATTTCAAGTAACACAACTGAATCCAACAAAAAATATGGTGCAAGTAGTGAAAAAATGGCTGCTGCCTACGCTGCTTTTGCTAATGGTGGTATTTATCACAAACCAATG

19F ST320 (3) *pbp1a* GGTCTAGATAGAGCTAAAACCTTCCTTAATGGTCTTGGTATCGACTATCCAAGCATGCATTATGCAAACGCCATTTCAAGTAACACAACTGAATCCAACAAAAAATATGGTGCAAGTAGTGAAAAAATGGCTGCTGCCTACGCTGCTTTTGCTAATGGTGGTATTTATCACAAACCAATG

19A ST320 (1) *pbp1a* GGTCTAGATAGAGCTAAAACCTTCCTTAATGGTCTTGGTATCGACTATCCAAGCATGCATTATGCAAACGCCATTTCAAGTAACACAACTGAATCCAACAAAAAATATGGTGCAAGTAGTGAAAAAATGGCTGCTGCCTACGCTGCTTTTGCTAATGGTGGTATTTATCACAAACCAATG

19A ST320 (2) *pbp1a* GGTCTAGATAGAGCTAAAACCTTCCTTAATGGTCTTGGTATCGACTATCCAAGCATGCATTATGCAAACGCCATTTCAAGTAACACAACTGAATCCAACAAAAAATATGGTGCAAGTAGTGAAAAAATGGCTGCTGCCTACGCTGCTTTTGCTAATGGTGGTATTTATCACAAACCAATG

19A ST320 (3) *pbp1a* GGTCTAGATAGAGCTAAAACCTTCCTTAATGGTCTTGGTATCGACTATCCAAGCATGCATTATGCAAACGCCATTTCAAGTAACACAACTGAATCCAACAAAAAATATGGTGCAAGTAGTGAAAAAATGGCTGCTGCCTACGCTGCTTTTGCTAATGGTGGTATTTATCACAAACCAATG

** ** * * ** ** ** ***** ******** ** ******** ***** * ** ** **** ******************** ** ** ************* ************** *********** ******** ***** ******** * *** * *********

19A ST199 (1) *pbp1a* TATATCCATAAAGTCGTCTTTAGTGATGGGAGTGAAAAAGAGTTCTCTAATGTCGGAACTCGTGCCATGAAGGAAACGACAGCCTATATGATGACCGACATGATGAAAACTGTCTTAGTATACGGAATCGGACGTGGAGCCTACCTACCTTGGCTTCCACAAGCAGGTAAAACAGGTACT

19A ST199 (2) *pbp1a* TATATCCATAAAGTCGTCTTTAGTGATGGGAGTGAAAAAGAGTTCTCTAATGTCGGAACTCGTGCCATGAAGGAAACGACAGCCTATATGATGACCGACATGATGAAAACTGTCTTAGTATACGGAATCGGACGTGGAGCCTACCTACCTTGGCTTCCACAAGCAGGTAAAACAGGTACT

19F ST320 (1) *pbp1a* TATATCAATAAAATCGTCTTTAGTGATGGTAGCGAAAAAGAATTTTCTGATGCTGGTACACGAGCTATGAAAGAGACTACTGCCTATATGATGACTGAAATGATGAAAACTGTTTTAACTTACGGAACAGGACGTGGAGCCTACCTACCATGGCTTCCACAAGCAGGTAAGACAGGTACT

19F ST320 (2) *pbp1a* TATATCAATAAAATCGTCTTTAGTGATGGTAGCGAAAAAGAATTTTCTGATGCTGGTACACGAGCTATGAAAGAGACTACTGCCTATATGATGACTGAAATGATGAAAACTGTTTTAACTTACGGAACAGGACGTGGAGCCTACCTACCATGGCTTCCACAAGCAGGTAAGACAGGTACT

19F ST320 (3) *pbp1a* TATATCAATAAAATCGTCTTTAGTGATGGTAGCGAAAAAGAATTTTCTGATGCTGGTACACGAGCTATGAAAGAGACTACTGCCTATATGATGACTGAAATGATGAAAACTGTTTTAACTTACGGAACAGGACGTGGAGCCTACCTACCATGGCTTCCACAAGCAGGTAAGACAGGTACT

19A ST320 (1) *pbp1a* TATATCAATAAAATCGTCTTTAGTGATGGTAGCGAAAAAGAATTTTCTGATGCTGGTACACGAGCTATGAAAGAGACTACTGCCTATATGATGACTGAAATGATGAAAACTGTTTTAACTTACGGAACAGGACGTGGAGCCTACCTACCATGGCTTCCACAAGCAGGTAAGACAGGTACT

19A ST320 (2) *pbp1a* TATATCAATAAAATCGTCTTTAGTGATGGTAGCGAAAAAGAATTTTCTGATGCTGGTACACGAGCTATGAAAGAGACTACTGCCTATATGATGACTGAAATGATGAAAACTGTTTTAACTTACGGAACAGGACGTGGAGCCTACCTACCATGGCTTCCACAAGCAGGTAAGACAGGTACT

19A ST320 (3) *pbp1a* TATATCAATAAAATCGTCTTTAGTGATGGTAGCGAAAAAGAATTTTCTGATGCTGGTACACGAGCTATGAAAGAGACTACTGCCTATATGATGACTGAAATGATGAAAACTGTTTTAACTTACGGAACAGGACGTGGAGCCTACCTACCATGGCTTCCACAAGCAGGTAAGACAGGTACT

****** ***** **************** ** ******** ** *** *** ** ** ** ** ***** ** ** ** ************** ** ************** *** ******* ******************** ******************** *********

19A ST199 (1) *pbp1a* TCTAACTATACTGACGAAGAAATTGAAAAGTATATCAAGAACACTGGTTACGTAGCTCCAGATGAAATGTTTGTAGGGTATACCCGCAAATATGCAATGGCTGTATGGACAGGCTATTCTAACCGTCTGACACCACTTGTAGGCGATGGCCTTACGGTCGCTGCTAAAGTTTACCGCTCT

19A ST199 (2) *pbp1a* TCTAACTATACTGACGAAGAAATTGAAAAGTATATCAAGAACACTGGTTACGTAGCTCCAGATGAAATGTTTGTAGGGTATACCCGCAAATATGCAATGGCTGTATGGACAGGCTATTCTAACCGTCTGACACCACTTGTAGGCGATGGCCTTACGGTCGCTGCTAAAGTTTACCGCTCT

19F ST320 (1) *pbp1a* TCTAACTATACTGACGAAGAAATTGAAAAGTATATCAAGAACACTGGCTACGTAGCTCCAGATGAAATGTTTGTGGGTTATACTCGTAAGTATTCTATGGCTGTATGGACAGGTTATTCGAATCGTTTAACTCCTATCGTTGGAGATGGTTTCCTAGTTGCAGCTAAAGTTTATCGCTCT

19F ST320 (2) *pbp1a* TCTAACTATACTGACGAAGAAATTGAAAAGTATATCAAGAACACTGGCTACGTAGCTCCAGATGAAATGTTTGTGGGTTATACTCGTAAGTATTCTATGGCTGTATGGACAGGTTATTCGAATCGTTTAACTCCTATCGTTGGAGATGGTTTCCTAGTTGCAGCTAAAGTTTATCGCTCT

19F ST320 (3) *pbp1a* TCTAACTATACTGACGAAGAAATTGAAAAGTATATCAAGAACACTGGCTACGTAGCTCCAGATGAAATGTTTGTGGGTTATACTCGTAAGTATTCTATGGCTGTATGGACAGGTTATTCGAATCGTTTAACTCCTATCGTTGGAGATGGTTTCCTAGTTGCAGCTAAAGTTTATCGCTCT

19A ST320 (1) *pbp1a* TCTAACTATACTGACGAAGAAATTGAAAAGTATATCAAGAACACTGGCTACGTAGCTCCAGATGAAATGTTTGTGGGTTATACTCGTAAGTATTCTATGGCTGTATGGACAGGTTATTCGAATCGTTTAACTCCTATCGTTGGAGATGGTTTCCTAGTTGCAGCTAAAGTTTATCGCTCT

19A ST320 (2) *pbp1a* TCTAACTATACTGACGAAGAAATTGAAAAGTATATCAAGAACACTGGCTACGTAGCTCCAGATGAAATGTTTGTGGGTTATACTCGTAAGTATTCTATGGCTGTATGGACAGGTTATTCGAATCGTTTAACTCCTATCGTTGGAGATGGTTTCCTAGTTGCAGCTAAAGTTTATCGCTCT

19A ST320 (3) *pbp1a* TCTAACTATACTGACGAAGAAATTGAAAAGTATATCAAGAACACTGGCTACGTAGCTCCAGATGAAATGTTTGTGGGTTATACTCGTAAGTATTCTATGGCTGTATGGACAGGTTATTCGAATCGTTTAACTCCTATCGTTGGAGATGGTTTCCTAGTTGCAGCTAAAGTTTATCGCTCT

*********************************************** ************************** ** ***** ** ** *** * ***************** ***** ** *** * ** ** * ** ** ***** * ** ** *********** ******

19A ST199 (1) *pbp1a* ATGATGACCTACCTGTCTGAAGGAAGCAATCCAGAAGATTGGAATATACCAGAGGGGCTCTACAGAAATGGAGAATTCGTATTTAAAAATGGTGCTCGTTCTACGTGGAACTCACCTGCTCCACAACAACCCCCATCAACTGAAAGTTCAAGCTCATCATCAGATAGTTCAACTTCACAG

19A ST199 (2) *pbp1a* ATGATGACCTACCTGTCTGAAGGAAGCAATCCAGAAGATTGGAATATACCAGAGGGGCTCTACAGAAATGGAGAATTCGTATTTAAAAATGGTGCTCGTTCTACGTGGAACTCACCTGCTCCACAACAACCCCCATCAACTGAAAGTTCAAGCTCATCATCAGATAGTTCAACTTCACAG

19F ST320 (1) *pbp1a* ATGATGACCTACCTGTCTGAAGGAAGCAATCCAGAGGATTGGAATATACCAGAGGGGCTCTACAGAAATGGAGAATTCGTATTTAAAAATGGTGCTCGTTCTACGTGGAGCTCACCTGCTCCACAACAACCCCCATCAACTGAAAGTTCAAGCTCATCATCAGATAGTTCAACTTCACAG

19F ST320 (2) *pbp1a* ATGATGACCTACCTGTCTGAAGGAAGCAATCCAGAGGATTGGAATATACCAGAGGGGCTCTACAGAAATGGAGAATTCGTATTTAAAAATGGTGCTCGTTCTACGTGGAGCTCACCTGCTCCACAACAACCCCCATCAACTGAAAGTTCAAGCTCATCATCAGATAGTTCAACTTCACAG

19F ST320 (3) *pbp1a* ATGATGACCTACCTGTCTGAAGGAAGCAATCCAGAGGATTGGAATATACCAGAGGGGCTCTACAGAAATGGAGAATTCGTATTTAAAAATGGTGCTCGTTCTACGTGGAGCTCACCTGCTCCACAACAACCCCCATCAACTGAAAGTTCAAGCTCATCATCAGATAGTTCAACTTCACAG

19A ST320 (1) *pbp1a* ATGATGACCTACCTGTCTGAAGGAAGCAATCCAGAGGATTGGAATATACCAGAGGGGCTCTACAGAAATGGAGAATTCGTATTTAAAAATGGTGCTCGTTCTACGTGGAGCTCACCTGCTCCACAACAACCCCCATCAACTGAAAGTTCAAGCTCATCATCAGATAGTTCAACTTCACAG

19A ST320 (2) *pbp1a* ATGATGACCTACCTGTCTGAAGGAAGCAATCCAGAGGATTGGAATATACCAGAGGGGCTCTACAGAAATGGAGAATTCGTATTTAAAAATGGTGCTCGTTCTACGTGGAGCTCACCTGCTCCACAACAACCCCCATCAACTGAAAGTTCAAGCTCATCATCAGATAGTTCAACTTCACAG

19A ST320 (3) *pbp1a* ATGATGACCTACCTGTCTGAAGGAAGCAATCCAGAGGATTGGAATATACCAGAGGGGCTCTACAGAAATGGAGAATTCGTATTTAAAAATGGTGCTCGTTCTACGTGGAGCTCACCTGCTCCACAACAACCCCCATCAACTGAAAGTTCAAGCTCATCATCAGATAGTTCAACTTCACAG

*********************************** ************************************************************************* *********************************************************************

19A ST199 (1) *pbp1a*  TCTAGCTCAACCACTCCAAGCACAAATAATAGTACGACTACCAATCCTAACAATAATACGCAACAATCAAATACAACCCCTGATCAACAAAATCAGAATCCTCAACCAGCACAACCAT

19A ST199 (2) *pbp1a*  TCTAGCTCAACCACTCCAAGCACAAATAATAGTACGACTACCAATCCTAACAATAATACGCAACAATCAAATACAACCCCTGATCAACAAAATCAGAATCCTCAACCAGCACAACCAT

19F ST320 (1) *pbp1a*  TCTAGCTCAACCACTCCAAGCACAAATAATAGTACGACTACCAATCCTAACAATAATACGCAACAATCAAATACAACCCCTGATCAACAAAATCAGAATCCTCAACCAGCACAACCAT

19F ST320 (2) *pbp1a*  TCTAGCTCAACCACTCCAAGCACAAATAATAGTACGACTACCAATCCTAACAATAATACGCAACAATCAAATACAACCCCTGATCAACAAAATCAGAATCCTCAACCAGCACAACCAT

19F ST320 (3) *pbp1a*  TCTAGCTCAACCACTCCAAGCACAAATAATAGTACGACTACCAATCCTAACAATAATACGCAACAATCAAATACAACCCCTGATCAACAAAATCAGAATCCTCAACCAGCACAACCAT

19A ST320 (1) *pbp1a*  TCTAGCTCAACCACTCCAAGCACAAATAATAGTACGACTACCAATCCTAACAATAATACGCAACAATCAAATACAACCCCTGATCAACAAAATCAGAATCCTCAACCAGCACAACCAT

19A ST320 (2) *pbp1a*  TCTAGCTCAACCACTCCAAGCACAAATAATAGTACGACTACCAATCCTAACAATAATACGCAACAATCAAATACAACCCCTGATCAACAAAATCAGAATCCTCAACCAGCACAACCAT

19A ST320 (3) *pbp1a*  TCTAGCTCAACCACTCCAAGCACAAATAATAGTACGACTACCAATCCTAACAATAATACGCAACAATCAAATACAACCCCTGATCAACAAAATCAGAATCCTCAACCAGCACAACCAT

**********************************************************************************************************************

19F_320_4 upstream *pbp1a* CTTTGATTAAGCGAGTGTTCTAATATAATTATAAGCGCCCTGTCATTACCGAACCCATTCGCCATTATAGTTGAC-GTAGTAGCCATCTACGGTCGTATTCACCGCCAAAGCACCTGAGCTATAAGCATAGTACCATTTGCCATTGACCTGGAACCAACCTGTCTTCATGTCTCCATTAC

19A_320_4 upstream *pbp1a* CTTTGATTAAGCGAGTGTTCTAATATAATTATAAGCGCCCTGTCATTACCGAACCCATTCGCCATTATAGTTGAC-GTAGTAGCCATCTACGGTCGTATTCACCGCCAAAGCACCTGAGCTATAAGCATAGTACCATTTGCCATTGACCTGGAACCAACCTGTCTTCATGTCTCCATTAC

19A_320_3 upstream *pbp1a* CTTTGATTAAGCGAGTGTTCTAATATAATTATAAGCGCCCTGTCATTACCGAACCCATTCGCCATTATAGTTGAC-GTAGTAGCCATCTACGGTCGTATTCACCGCCAAAGCACCTGAGCTATAAGCATAGTACCATTTGCCATTGACCTGGAACCAACCTGTCTTCATGTCTCCATTAC

19F_320_1 upstream *pbp1a* CTTTGATTAAGCGAGTGTTCTAATATAATTATAAGCGCCCTGTCATTACCGAACCCATTCGCCATTATAGTTGAC-GTAGTAGCCATCTACGGTCGTATTCACCGCCAAAGCACCTGAGCTATAAGCATAGTACCATTTGCCATTGACCTGGAACCAACCTGTCTTCATGTCTCCATTAC

19F_320_3 upstream *pbp1a* CTTTGATTAAGCGAGTGTTCTAATATAATTATAAGCGCCCTGTCATTACCGAACCCATTCGCCATTATAGTTGAC-GTAGTAGCCATCTACGGTCGTATTCACCGCCAAAGCACCTGAGCTATAAGCATAGTACCATTTGCCATTGACCTGGAACCAACCTGTCTTCATGTCTCCATTAC

19A_199_4 upstream *pbp1a* CTTTGATTAAGCGAGTGTTCTAATATAATTATAAGCGCCCTGTCATTACCGAACCCATTCGCCATTATAGTTGACAGAA-TAGCCATCTACGGTCGTATTCACTGCCAAAGCACCTGAGCTATAAGCATAGTACCAGTTGCCATTGACCTGGAACCAACCTGTCTTCATGTCTCCATTAC

19A_199_3 upstream *pbp1a* CTTTGATTAAGCGAGTGTTCTAATATAATTATAAGCGCCCTGTCATTACCGAACCCATTCGCCATTATAGTTGACAGAA-TAGCCATCTACGGTCGTATTCACTGCCAAAGCACCTGAGCTATAAGCATAGTACCAGTTGCCATTGACCTGGAACCAACCTGTCTTCATGTCTCCATTAC

19A_199_1 upstream *pbp1a* CTTTGATTAAGCGAGTGTTCTAATATAATTATAAGCGCCCTGTCATTACCGAACCCATTCGCCATTATAGTTGACAGAA-TAGCCATCTACGGTCGTATTCACTGCCAAAGCACCTGAGCTATAAGCATAGTACCAGTTGCCATTGACCTGGAACCAACCTGTCTTCATGTCTCCATTAC

*************************************************************************** * * *********************** ******************************** *******************************************

19F_320_4 upstream *pbp1a* CTGCATTTAGGTAGTACCAAGTTAAACCATCTTGATACCAGCCAGTTACCATTGCTCCTGATGAACGGAGGTAGTACCACTTGTTACCAAGGTATTGCCATCCTGTTTTCATATCACCATTTGGCTGGTCTAAATAATACCAAGTGGTACCTTCCTGATACCAGCCAGTGGCCATTGCTC

19A_320_4 upstream *pbp1a* CTGCATTTAGGTAGTACCAAGTTAAACCATCTTGATACCAGCCAGTTACCATTGCTCCTGATGAACGGAGGTAGTACCACTTGTTACCAAGGTATTGCCATCCTGTTTTCATATCACCATTTGGCTGGTCTAAATAATACCAAGTGGTACCTTCCTGATACCAGCCAGTGGCCATTGCTC

19A_320_3 upstream *pbp1a* CTGCATTTAGGTAGTACCAAGTTAAACCATCTTGATACCAGCCAGTTACCATTGCTCCTGATGAACGGAGGTAGTACCACTTGTTACCAAGGTATTGCCATCCTGTTTTCATATCACCATTTGGCTGGTCTAAATAATACCAAGTGGTACCTTCCTGATACCAGCCAGTGGCCATTGCTC

19F_320_1 upstream *pbp1a* CTGCATTTAGGTAGTACCAAGTTAAACCATCTTGATACCAGCCAGTTACCATTGCTCCTGATGAACGGAGGTAGTACCACTTGTTACCAAGGTATTGCCATCCTGTTTTCATATCACCATTTGGCTGGTCTAAATAATACCAAGTGGTACCTTCCTGATACCAGCCAGTGGCCATTGCTC

19F_320_3 upstream *pbp1a* CTGCATTTAGGTAGTACCAAGTTAAACCATCTTGATACCAGCCAGTTACCATTGCTCCTGATGAACGGAGGTAGTACCACTTGTTACCAAGGTATTGCCATCCTGTTTTCATATCACCATTTGGCTGGTCTAAATAATACCAAGTGGTACCTTCCTGATACCAGCCAGTGGCCATTGCTC

19A_199_4 upstream *pbp1a* CTGCATTTAGGTAGTACCAAGTTGAACCATCTTGATACCAACCAGTTGCCATAGCTCCTGATGAACGGAGATAGTACCATTTGTTCCCAAGGTTTTGCCAACCTGTTTTCATATCGCCATTTGGGTGGTCTAAATAATACCAAGTGGTACCTTCCTGATACCAGCCAGTGGCCATTGCTC

19A_199_3 upstream *pbp1a* CTGCATTTAGGTAGTACCAAGTTGAACCATCTTGATACCAACCAGTTGCCATAGCTCCTGATGAACGGAGATAGTACCATTTGTTCCCAAGGTTTTGCCAACCTGTTTTCATATCGCCATTTGGGTGGTCTAAATAATACCAAGTGGTACCTTCCTGATACCAGCCAGTGGCCATTGCTC

19A_199_1 upstream *pbp1a* CTGCATTTAGGTAGTACCAAGTTGAACCATCTTGATACCAACCAGTTGCCATAGCTCCTGATGAACGGAGATAGTACCATTTGTTCCCAAGGTTTTGCCAACCTGTTTTCATATCGCCATTTGGGTGGTCTAAATAATACCAAGTGGTACCTTCCTGATACCAGCCAGTGGCCATTGCTC

*********************** **************** ****** **** ***************** ******** ***** ******* ****** ************** ******** *******************************************************

19F_320_4 upstream *pbp1a* CTGATGAACGGAGGTAGTACCAGTTATTACCTAGATATTGCCATCCTGTTTG -----------------TCATTACCGAACCCATTCGCCATTATAGTTGAC-GTAGTAGCCATCTACGGTCGTATTCACTGCCAAGGCACCTGAGCTATAAGCGTAGTACCA-TCTGCCAT

19A_320_4 upstream *pbp1a* CTGATGAACGGAGGTAGTACCAGTTATTACCTAGATATTGCCATCCTGTTTG ATTCACAATCGCTTCTTTCATTATTGAGCCCATTCGCCATTATAGTTGAC-GTAGTAGCCATCTACGGTCGTATTCACTGCCAAGGCACCTGAGCTATAAGCGTAGTACCA-TCTGCCAT

19A_320_3 upstream *pbp1a* CTGATGAACGGAGGTAGTACCAGTTATTACCTAGATATTGCCATCCTGTTTG ATTCACAATCGCTTCTTTCATTATTGAGCCCATTCGCCATTATAGTTGAC-GTAGTAGCCATCTACGGTCGTATTCACTGCCAAGGCACCTGAGCTATAAGCGTAGTACCA-TCTGCCAT

19F_320_1 upstream *pbp1a* CTGATGAACGGAGGTAGTACCAGTTATTACCTAGATATTGCCATCCTGTTTG -----------------TCATTACCGAACCCATTCGCCATTATAGTTGAC-GTAGTAGCCATCTACGGTCGTATTCACTGCCAAGGCACCTGAGCTATAAGCGTAGTACCA-TCTGCCAT

19F_320_3 upstream *pbp1a* CTGATGAACGGAGGTAGTACCAGTTATTACCTAGATATTGCCATCCTGTTTG -----------------TCATTACCGAACCCATTCGCCATTATAGTTGAC-GTAGTAGCCATCTACGGTCGTATTCACTGCCAAGGCACCTGAGCTATAAGCGTAGTACCA-TCTGCCAT

19A_199_4 upstream *pbp1a* CTGAGGAACGGAGGTAGTACCAGTTATTACCTAGATATTGCCAACCTGTTTG ATT-ACAAGCGC-CCTGTCATTACCGAACCCATTCGCCATTATAGTTGACAG-AATAGCCATCTACGGTCGTATTCACTGCCAAAGCACCTGAGCTATAAGCATAGTACCAGT-TGCCAT

19A_199_3 upstream *pbp1a* CTGAGGAACGGAGGTAGTACCAGTTATTACCTAGATATTGCCAACCTGTTTG ATT-ACAAGCGC-CCTGTCATTACCGAACCCATTCGCCATTATAGTTGACAG-AATAGCCATCTACGGTCGTATTCACTGCCAAAGCACCTGAGCTATAAGCATAGTACCAGT-TGCCAT

19A_199_1 upstream *pbp1a* CTGAGGAACGGAGGTAGTACCAGTTATTACCTAGATATTGCCAACCTGTTTG ATT-ACAAGCGC-CCTGTCATTACCGAACCCATTCGCCATTATAGTTGACAG-AATAGCCATCTACGGTCGTATTCACTGCCAAAGCACCTGAGCTATAAGCATAGTACCAGT-TGCCAT

**** ************************************** ******** ****** ** ********************** * * ***************************** ***************** ******** * ******

19F_320_4 upstream *pbp1a* TGACCTGGAACCAACCTGTCGTCAT

**N.B: The black arrows demarcate an area of duplication in SPN MDR 19A ST320.**

19A_320_4 upstream *pbp1a* TGACCTGGAACCAACCTGTCGTCAT

19A_320_3 upstream *pbp1a* TGACCTGGAACCAACCTGTCGTCAT

19F_320_1 upstream *pbp1a* TGACCTGGAACCAACCTGTCGTCAT

19F_320_3 upstream *pbp1a* TGACCTGGAACCAACCTGTCGTCAT

19A_199_4 upstream *pbp1a* TGACCTGGAACCAACCTGTCTTCAT

19A_199_3 upstream *pbp1a* TGACCTGGAACCAACCTGTCTTCAT

19A_199_1 upstream *pbp1a* TGACCTGGAACCAACCTGTCTTCAT

******************** ****
